# Hydrocolloid–Nanomaterial Composite Films: Preservation Performance, Preparation Method and Sustainable Development

**DOI:** 10.3390/foods15040685

**Published:** 2026-02-13

**Authors:** Lin Meng, Cheng Peng, Linling Li, Yingtang Lu, Hua Cheng

**Affiliations:** 1 School of Modern Industry for Selenium Science and Engineering, Wuhan Polytechnic University, Wuhan 430048, China; mengl021@126.com (L.M.); pengcheng0612@126.com (C.P.); yingtlu@whu.edu.cn (Y.L.); 2 National R&D Center for Se-Rich Agricultural Products Processing, Wuhan Polytechnic University, Wuhan 430023, China

**Keywords:** nanomaterials, preparation method, preservation performance, antioxidant, antimicrobial

## Abstract

Traditional plastic preservation films face significant environmental challenges due to their non-degradable nature and limited functional versatility. In contrast, hydrocolloid–nanomaterial composite films—which integrate biopolymer matrices (e.g., cellulose, chitosan, alginate and gelatin) with nanoparticles such as SiO_2_, Se, TiO_2_, or ZnO—have emerged as a prominent research focus. These composite films preserve the inherent biodegradability and biocompatibility of hydrocolloids, while the nanomaterials, when stably dispersed, enhance interfacial interactions through electrostatic forces, hydrogen-bonding, or coordination bonds. This synergy endows the films with multifunctional properties, including antimicrobial activity, antioxidant capacity, UV-shielding performance, and stimuli-responsive intelligence. Prepared via techniques like electrospinning, solution casting, reactive extrusion, and coating, they exhibit excellent mechanical strength, barrier properties, and multifunctionality, effectively extending the shelf life of fruits, vegetables, meats, etc. However, challenges remain: nanomaterial dispersion, migration risks, and scalable production. This review summarizes recent advances to guide green preparation optimization, balance performance and safety, and advance sustainable development in food packaging.

## 1. Introduction

Food preservation films are indispensable packaging materials in the food industry [1]. Their core functions are centered on extending the shelf life of foods through three parallel mechanisms: physical barrier formation [2], microbial growth inhibition [3] and oxidative reaction retardation [4]. Growing evidence demonstrates that preservation films can also partially maintain the nutritional components of foods. For example, they mitigate the oxidation of vitamins and soluble solids, preserving the nutritional value of foods [5]. In terms of food safety, preservation films complying with relevant regulatory standards effectively prevent external contamination and mitigate potential safety hazards during storage and transportation, thereby providing consumers with safer and healthier food choices.

Though widely used, traditional plastic preservation films—such as polyethylene (PE), polypropylene (PP), and polyvinyl chloride (PVC)—are produced via petroleum-based cracking and polymerization and face increasing limitations [6]. First, their non-degradability gives rise to “white pollution,” exacerbating ecological pressures [7]. Globally, around 300 million tons of plastic waste are generated each year, with food packaging accounting for more than 40% [8]. In the absence of policy interventions, an estimated 1.3 billion metric tons of plastic waste could enter natural ecosystems by 2040 [9]. Second, traditional films rely solely on physical barriers, lacking active antimicrobial, antioxidant, or smart monitoring capabilities, which limit their effectiveness in complex storage environments, cold-chain logistics, or high-humidity conditions. Furthermore, some plasticizers or synthetic preservatives in these films pose potential migration risks, thereby raising concerns regarding food safety.

**Figure 1:**
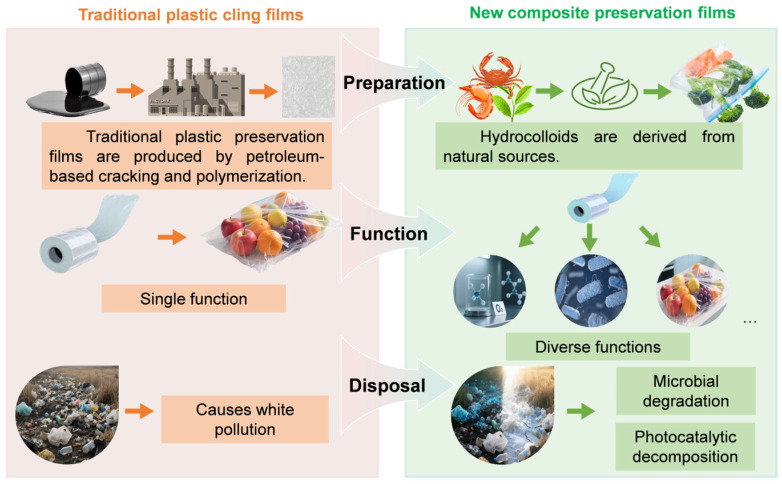
Comparison of traditional plastic cling film and new composite preservation films in terms of synthesis, functionality, and environmental impact.

To address these challenges, hydrocolloid matrix-based preservation films have emerged as a prominent research focus, primarily attributed to their unique advantages. First, hydrocolloid matrices (e.g., chitosan, gelatin, sodium alginate) are derived from natural sources and can be degraded via microbial or photocatalytic pathways, thereby reducing their environmental impacts [10]. With rising consumer demands for food safety, quality, and extended shelf life, coupled with the growing market requirements for advanced food packaging solutions, solely relying on the physical barrier function of hydrocolloid matrices has been demonstrated to be inadequate. To endow preservation films with enhanced comprehensive properties, nanomaterial-based composite modification has been identified as an effective strategy. The synergistic effect between hydrocolloid matrices and nanomaterials endows the films with enhanced multifunctional properties. Specifically, nanomaterials (e.g., selenium nanoparticles [SeNPs], TiO_2_, ZnO) markedly enhance antimicrobial activity when incorporated into hydrocolloid matrices [11,12,13]. Certain composite films further exhibit smart responsive capabilities; for instance, pH-sensitive nanoparticles (e.g., anthocyanins) allow for the real-time monitoring of food freshness through colorimetric changes [14]. Moreover, compared with conventional films, these composite films demonstrate superior mechanical and barrier properties [15]. Nanomaterials such as cellulose nanocrystals and nanoclays can mitigate structural defects in polysaccharide-based films. Through formulation optimization, these composite materials meet the diverse preservation needs of fruits (e.g., strawberries, mangoes) and high-protein foods (e.g., beef, fish), highlighting their extensive application prospects (Figure 1).

**Figure 2:**
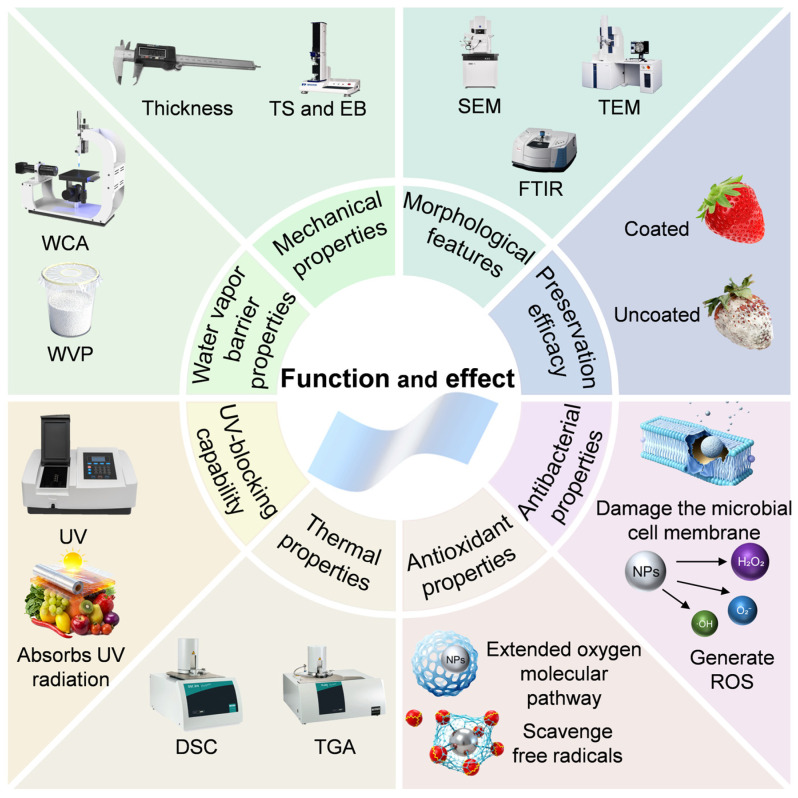
Function and effect of hydrocolloid–nanomaterial composite films.

In recent years, research on hydrocolloid matrix–nanomaterial composite films has intensified, leading to notable breakthroughs. Zhang et al. evaluated the functional characteristics of silica nanoparticles (SiO_2_ NPs) in biodegradable polysaccharide-based composite films. Their study revealed that SiO_2_ NPs enhanced the mechanical properties of chitosan and sodium alginate films. Leveraging their mesoporous structure, SiO_2_ NPs can also load bioactive agents such as clove oil and curcumin for sustained antimicrobial release, effectively inhibiting *Escherichia coli* (*E. coli*) and *Staphylococcus aureus* (*S. aureus*). This modification extended the shelf life of strawberries to 7 days, reduced weight loss by 50%, and preserved their total phenolic content [16]. Yu et al. developed a self-reinforced multifunctional starch nanocomposite film by incorporating quaternized chitosan and epigallocatechin gallate (EGCG)-modified SeNPs into starch through a facile aqueous mixing method. The film exhibited excellent antioxidant, antimicrobial and UV-shielding capabilities, alongside biodegradability and biosafety, effectively prolonging the shelf life of postharvest litchis [17]. Another study integrated TiO_2_ nanotube arrays into a composite matrix composed of soy protein isolate and carboxymethyl chitosan matrix, fabricating a composite film with higher tensile strength (TS), improved water barrier properties and uncompromised biodegradability, which preserved the quality of blueberries and extended their shelf life [18]. Guerra et al. innovatively isolated pectin from the peels of Brazilian *Caryocar brasiliense* fruits and incorporated ZnONPs to fabricate films that achieved complete degradation in soil within two days, thereby alleviating environmental pollution [19]. Tran et al. employed gamma-ray irradiation to reduce selenate to SeNPs, which were then combined with sodium alginate to develop an edible coating. This coating inhibited the growth of *Neopestalotiopsis sp.* and *Fusarium oxysporum*, reduced weight loss and decay in strawberries stored at room temperature, preserved their firmness, natural appearance, and content of vitamins C and B9, and enhanced selenium content, thereby extending shelf life [20]. Against the backdrop of tightening global environmental regulations and growing consumer preference for sustainable packaging [21], composite preservation films exhibit enormous application potential owing to their biodegradability and biosafety. These films cater to diverse food preservation requirements in various food categories, including fruits, vegetables, meats, seafood and dairy products, through customized solutions [22]. Hydrocolloid–nanomaterial composite films, characterized by their eco-friendly nature and superior preservation efficacy, are well-positioned for widespread application and market expansion (Figure 2).

This review systematically synthesizes the progress in research into hydrocolloid matrix (e.g., chitosan, gelatin, sodium alginate, cellulose derivatives, starch) and nanomaterial (e.g., Se, TiO_2_, ZnO) composite films. Particular emphasis is placed on the intrinsic characteristics, modification strategies, preparation technologies and interaction mechanisms of hydrocolloid matrices and nanomaterials. Furthermore, this review discusses the prospects and challenges of hydrocolloid–nanomaterial composite films in commercial applications, aiming to provide a theoretical basis and technical references for the development of next-generation degradable smart food packaging.

## 2. Hydrocolloid Matrix

### 2.1. Chitosan

Chitosan, a natural linear polysaccharide obtained via the deacetylation of chitin, consists of D-glucosamine and N-acetyl-D-glucosamine linked by β-1,4-glycosidic bonds [23]. As a food preservation film material, chitosan exhibits unique advantages. Its abundant availability and biodegradability effectively mitigate “white pollution” induced by plastic packaging. Chitosan disrupts the structure of bacterial cell membranes, conferring inherent antimicrobial properties that extend food shelf life [24]. Additionally, its antioxidant capacity inhibits lipid oxidation and delays fruit browning [25]. However, pure chitosan films exhibit inherent limitations, including inadequate flexibility, high water solubility, and poor mechanical properties. Recent studies have focused on incorporating chitosan with other polymers to develop innovative composite materials with enhanced mechanical strength and barrier efficiency. For example, Liu et al. grafted chitosan onto bacterial cellulose via Schiff base reactions and incorporated curcumin to fabricate a bio-composite film with exceptional antioxidant activity, significantly preserving the quality of strawberries and edible oils [26]. Although chitosan/cellulose composite films exhibit biocompatibility and antimicrobial activity, their mechanical performance and hydrophobicity still need further enhancement. Beji et al. innovatively incorporated aluminum chloride (AlCl_3_) as a filler into the chitosan/cellulose matrix to fabricate a ternary chitosan/AlCl_3_/cellulose composite film [27]. This film demonstrated improved mechanical properties, hydrophobicity, antimicrobial activity and UV-shielding capability, providing novel insights for the development of high-performance bioactive packaging.

### 2.2. Gelatin

Gelatin, a water-soluble protein derived from the partial hydrolysis of animal collagen [28], is primarily sourced from pigskin, cattle bones, bovine hides and fish scales. Rich in repeating units of glycine, proline and hydroxyproline, gelatin exhibits high UV absorption capacity. Structurally analogous to collagen, gelatin forms a triple-helix structure consisting of three polypeptide chains, which endows it with favorable physical strength [29]. Due to its non-toxicity, biocompatibility, biodegradability and film-forming property, gelatin has been widely applied in food packaging [30]. However, pure gelatin films suffer from inherent drawbacks, including low mechanical strength, poor water barrier properties, and high microbial susceptibility, which limit their practical application in food preservation [31]. Blending gelatin with other hydrocolloid matrices or incorporating nanofillers represents an effective strategy to enhance the mechanical and barrier properties of gelatin-based films. For instance, Sun et al. developed resveratrol-loaded gelatin/carboxymethyl cellulose edible active films using three methods, which exhibited improved hydrophobicity and antioxidant activity while effectively inhibiting the oxidation of soybean oil [32]. Xu et al. incorporated quercetin-loaded zein into gelatin films to fabricate a composite with superior UV-shielding and antimicrobial properties, extending the shelf life of strawberries to 8 days [33].

### 2.3. Sodium Alginate

Sodium alginate, a natural anionic polysaccharide extracted from brown algae (e.g., kelp, *Macrocystis*), is a linear copolymer composed of alternating β-D-mannuronic acid (M units) and α-L-guluronic acid (G units) linked via 1,4-glycosidic bonds [34]. Valued for its film-forming property, biocompatibility and excellent oxygen and oil barrier properties, sodium alginate has been widely applied in food preservation [35]. It prolongs the shelf life of food products through multiple mechanisms, including the formation of selective gas-permeable coatings that retard fruit respiration and oxidative processes [36], as well as synergistic effects with antimicrobial agents. For example, Riahi et al. fabricated a multifunctional composite film by incorporating red pepper-based carbon dots (RP-CDs) into a sodium alginate–gelatin matrix, which exhibited a UV-shielding efficiency of 99.1%, an ABTS radical scavenging activity of 100%, and an antimicrobial efficiency of 99.9% against *L. monocytogenes*, effectively extending the shelf life of grapes to 24 days [37]. To address the low mechanical strength and high hygroscopicity of pure sodium alginate films, researchers have developed multifunctional composite materials. Zhang et al. developed a carboxymethyl chitosan/sodium alginate/citric acid hydrogel film that can coat strawberries within 10 s, achieving a lethality rate of greater than 99% against *E. coli* and *S. aureus* while maintaining performance after five regeneration cycles [38]. Leveraging the pH-sensitive property of sodium alginate, Cao et al. developed an antioxidant and antimicrobial sodium alginate/chitosan-based smart bilayer film. The incorporation of TiO_2_ NPs stabilized the color of anthocyanins, enabling real-time visual monitoring of food quality [39].

### 2.4. Starch

Starch, a widely available natural polysaccharide derived from sources such as potatoes, corn, yams and cassava, consists of amylose (linear chains linked by α-1,4-glycosidic bonds) and amylopectin (branched chains with α-1,6-glycosidic bonds at branch points), forming a semi-crystalline granular structure [40]. The ratio of amylose to amylopectin varies with starch source, directly influencing physicochemical and film-forming properties. High-amylose starch yields films with superior mechanical strength and gas barrier performance [41], while high-amylopectin starch reduces water solubility and mechanical properties [42] but increases crystallinity [43]. Owing to its low cost, edibility, biocompatibility and film-forming ability, starch is an ideal candidate for biodegradable food packaging films [44]. Starch-based films undergo a staged degradation process: enzymes like amylase and glucanase cleave glycosidic bonds to generate glucose [45], which is further mineralized into CO_2_ and H_2_O by soil microorganisms [46]. Higher starch content accelerates the degradation rate; pure starch films degrade faster than blended films incorporating polyvinyl alcohol (PVA), nanoclays, or essential oils [47]. Leveraging these properties, starch can be modified via physical or chemical methods to meet practical requirements for film applications. Luo et al. employed a solution casting to prepare tea polyphenol/MgO nanoparticle-reinforced potato starch-based biodegradable nanocomposite films [48]. These films exhibited robust antioxidant and antimicrobial activity, effectively preserving the quality of bananas, strawberries and grapes by reducing water loss and respiration during storage.

Carboxymethyl Starch (CMS), an anionic polysaccharide derivative, is obtained by etherifying alkoxylated polysaccharides with chloroacetic acid (or its sodium salt) under alkaline conditions. Owing to its safety and non-toxicity, CMS is applicable in cosmetics and pharmaceuticals, and is included in the United States Pharmacopeia (USP) and British Pharmacopoeia (BP) [49]. Compared with the native starch, CMS has carboxymethyl groups (–CH_2_COONa) introduced into its molecular chain, which disrupts the intramolecular hydrogen bond network of starch granules, thereby significantly reducing its crystallinity and endowing it with new properties, including instant solubility in cold water, high viscosity, high water retention and good film-forming properties [50]. These favorable characteristics render CMS highly promising for applications in food preservation. For example, Xie et al. constructed a “steel wire mesh”-structured multifunctional fresh-keeping film, 2.5ZnO/10CMS/CS, through the coordinated cross-linking of ZnONPs, CMS and chitosan. This film exhibits a light transmittance of approximately 89% and an antimicrobial duration of over 202 h, effectively maintaining the freshness of strawberries for 5 days. More importantly, this film is humidity-sensitive and enables real-time monitoring of the storage environment [51] (Table 1).

### 2.5. Other Polysaccharide Materials

#### 2.5.1. Cellulose

Cellulose, the most abundant polysaccharide in nature, is predominantly found in plant cell walls and can also be biosynthesized by microorganisms [57]. Composed of glucose units linked by β-1,4-glycosidic bonds [58], cellulose exhibits high crystallinity and mechanical strength, attributed to its tightly packed molecular configuration, thereby forming dense barrier layers capable of obstructing oxygen, water vapor, and other gases and liquids [59]. Naturally water-insoluble, natural cellulose lacks the ability to disperse in aqueous media to form viscous colloidal solutions [60]. Through chemical modification, however, cellulose derivatives gain water solubility, or the ability to form stable colloidal dispersions in water, while exhibiting thickening, gelling and stabilizing properties, which render them highly suitable for applications in food preservation [61].

Carboxymethyl cellulose (CMC), a cellulose derivative, offers superior water solubility, film-forming ability and biocompatibility [62]. Compared with pure cellulose films, CMC-based films demonstrate enhanced flexibility, transparency, barrier properties and mechanical performance. Furthermore, their preservation efficacy can be further improved by incorporating additives such as chitosan or nanoparticles. For example, Parya et al. demonstrated that Cu–TiO_2_-doped CMC films exhibited significant antimicrobial and photocatalytic activity under visible light, effectively delaying banana browning and extending shelf life [52].

Hydroxypropyl methylcellulose is a water-soluble polymer derivative obtained by the etherification modification of natural cellulose, which is characterized by the partial substitution of hydroxyl groups in the glucose units of cellulose with methoxy groups (-OCH_3_) and hydroxypropoxy groups (-OCH_2_CH(OH)CH_3_) [63]. It has excellent film-forming properties and can be used as an emulsifier, thickener and stabilizer in the cosmetics, pharmaceutical and food industries [64]. Building on earlier work that enhanced the mechanical properties of hydroxypropyl methylcellulose with xanthan gum, Zheng et al. further incorporated biomass carbon quantum dots (CQDs) to fabricate a xanthan gum/hydroxypropyl methylcellulose/carbon quantum dots (XHC) composite film, which has the triple functions of biodegradability, high-efficiency antimicrobial activity and excellent freshness-keeping. The CQDs in the XHC composite film not only inhibit the proliferation of *Colletotrichum gloeosporioides* by inducing the apoptosis of the pathogen, but also effectively improve the UV-shielding performance of the film, further enhancing its freshness-keeping efficacy [65]. Furthermore, studies have shown that composite coatings based on hydroxypropyl methylcellulose loaded with black chokeberry extract and ZnONPs can completely inactivate the Φ6 bacteriophage, confirming their antiviral activity [66]. This unique property equips composite food preservation films with antiviral protective capabilities, which can not only extend the shelf life of fresh products and mitigate the risk of contamination but also establish a robust safeguard for consumers’ food biosafety, thereby exhibiting promising application prospects in the field of functional packaging.

#### 2.5.2. Pectin

Pectin, a heteropolysaccharide abundant in plant cell walls (e.g., citrus peels, apple pomace, banana peels) [67], varies in content and properties depending on its source [68]. Its backbone consists of α-1,4-linked galacturonic acid residues, with side-chain variations classifying it into types such as homogalacturonan (HG), rhamnogalacturonan-I (RG-I) and rhamnogalacturonan-II (RG-II) [67]. The degree of esterification (DE) is a critical parameter influencing pectin’s gelation behavior: high-DE pectin forms gels via hydrogen bonds and hydrophobic interactions, while low-DE pectin relies on calcium ion crosslinking [69]. Pectin-based films, characterized by full biodegradability and non-toxicity, have garnered growing attention for food packaging. By incorporating natural antimicrobials (e.g., tea polyphenols, nano-TiO_2_) or essential oils, these films effectively inhibit microbial growth and delay oxidation in fruits and vegetables. Guo et al. fabricated a composite film by integrating citrus peel-derived pectin, flavonoids and soy protein isolate, which extended the shelf life of red grapes and pork by demonstrating strong antimicrobial and antioxidant activity [53]. Li et al. developed a pectin film incorporating zein NPs and Fe^3+^-loaded polyphenol-enriched extract (PPE), achieving controlled PPE release, enhanced mechanical properties, and superior antioxidant/antimicrobial performance, thereby effectively preserving the quality of strawberries [54].

#### 2.5.3. Hyaluronic Acid

Hyaluronic acid (HA), a natural polysaccharide widely used in cosmetics and medicine [70], has recently garnered increasing attention in food preservation owing to its biodegradability [71], biocompatibility and inherent antioxidant properties [72]. Zhou et al. developed a multifunctional HA-based coating by incorporating cinnamaldehyde and 2-hydroxypropyl-β-cyclodextrin, which exhibited excellent antimicrobial and antioxidant effects, prolonging the shelf life of bananas and apples [55]. Leveraging HA’s antioxidant activity, Al-Hilifi et al. developed a polysaccharide–protein composite edible coating combining HA, chitosan and gelatin, effectively slowing strawberry ripening and decay [56]. These studies highlight HA’s potential in food preservation. However, future research should address challenges such as HA’s biocompatibility optimization and formulation stability to advance its practical application in food packaging.

## 3. Enhancement of Food Hydrocolloids by Nanomaterials

In recent years, nanomaterials have gained significant attention in food preservation and packaging. Nanoparticles such as ZnO, Ag, TiO_2_ and Se have shown remarkable potential in preservation films due to their unique physicochemical properties. In practical applications, nanomaterials enhance the mechanical, thermal, barrier and functional properties of preservation films through physical filling, chemical bonding and synergistic interactions. The functional achievements of different nanomaterials rely on their unique physicochemical properties and mechanisms of action, providing multiple options for the functional modification of fresh-keeping films. The antimicrobial properties of ZnONPs stem from a dual mechanism: on the one hand, they release Zn^2+^ to damage the microbial cell membrane and demonstrate antifungal activity, making them widely used for antimicrobial modification in food packaging. On the other hand, under the action of light, they can generate reactive oxygen species (ROS) such as O_2_^−^, ·OH and H_2_O_2_. This ROS generation pathway has been recognized as its key antimicrobial mechanism [73]. Silver nanoparticles (AgNPs) exhibit broad-spectrum antimicrobial properties [74], inhibiting bacterial proliferation through membrane disruption. TiO_2_-NPs possess photocatalytic activity, generating ROS under UV or visible light to degrade microbial membranes and organic compounds [75]. SeNPs, with low toxicity, high bioactivity, antioxidant capacity and antimicrobial effects, have emerged as promising candidates for food preservation, effectively scavenging free radicals and delaying oxidative deterioration [76,77,78]. Carbon Dots (CDs) are a novel nanomaterial with diameters of less than 10 nm [79] that have significant free radical scavenging ability, UV absorption and antimicrobial properties [80].

In summary, various nanomaterials exhibit distinct focuses in their core properties and action mechanisms, and their functions are based on their unique physicochemical properties that enable their participation in the regulation of the performance of food preservation films. In the following sections, the action modes and regulation effects of different nanomaterials will be elaborated in detail in relation to their mechanical, barrier and thermal properties in food preservation films (Table 2).

### 3.1. Mechanical Properties

Nanomaterials can enhance the mechanical strength of composite films through their high surface area and affinity with polymers [89]. For example, Zhang et al. developed a modified chitosan-based TiO_2_ nanoparticle–nisin composite film where TiO_2_-NPs embedded within the chitosan matrix formed a crystalline structure. This structure enhanced internal friction within the chitosan matrix and thereby enhanced the film’s TS [90]. This finding indicates that the physical filling effect of inorganic nanoparticles and the optimization of the matrix microstructure constitute an effective strategy for enhancing the mechanical properties of films, which is particularly applicable to polysaccharide-based matrices with a relatively loose intrinsic structure. In another study, Zhang et al. improved the mechanical properties of films by mixing SiO_2_ into CMC/Sodium alginate [81]. TS improved by 33.5% and elongation at break (EB) by 25.7% compared with SiO_2_-free films. This is attributed to the formation of a crosslinking network between the hydroxyl groups on SiO_2_ and the carboxyl groups on CMC/Sodium alginate. This study highlights the superiority of chemical crosslinking over mere physical filling: the constructed cross-linked network can effectively improve the stress transfer efficiency between polymer molecular chains, thereby achieving a synergistic enhancement of strength and ductility, which represents a crucial modification advantage for composite films requiring both structural stability and a certain degree of flexibility in practical applications. Notably, the mechanical reinforcement effect of multifunctional nanomaterials on films typically exhibits concentration-dependent characteristics [91], which is exemplified by the work of Guo et al. The study used *Sophora japonica* extract (95% rutin) as a single raw material to synthesize multifunctional carbon dots (R-CDs) with broadband ultraviolet absorption and robust antioxidant and antibacterial activities using a hydrothermal method. Upon the incorporation of R-CDs into the gelatin film matrix, the TS first increased and then decreased with 1% (*w*/*w*) R-CDs identified as the optimal concentration; meanwhile, the EB increased continuously, reaching 358% at a R-CD concentration of 1.5% (*w*/*w*) [88]. This phenomenon reveals two key points: First, that there is a concentration threshold for the interfacial compatibility between R-CDs and the gelatin matrix; excessive amounts will lead to the agglomeration of R-CDs, which in turn causes a decrease in tensile strength. Second, the continuous increase in elongation at break indicates that R-CDs can serve as flexible linkers between gelatin molecular chains, which is related to the small size and abundant surface functional groups of carbon dots.

### 3.2. Barrier Properties

The barrier properties of preservation films are critical for maintaining quality, as they help mitigate moisture loss and retard the oxidative deterioration of food [92]. Nanoparticles incorporated within the polymer matrix can lengthen the permeation pathways for water vapor and oxygen, thereby enhancing the film’s barrier performance. However, the structural defects of single nanomaterials often limit their modification effects, while composite nanomaterials can achieve performance optimization through component synergy. Kang et al. reported that, due to ZIF-67’s porous structure, when it was incorporated alone into CMC films, it acted as a rapid diffusion channel for water molecules. This resulted in a slight increase in the water vapor transmission rate (WVTR) from 0.41 to 0.44 compared with pure CMC films, thus causing a minor decline in water vapor barrier performance. In contrast, Ag@ZIF-67 composite nanomaterials physically blocked the pores of ZIF-67 via AgNPs, which directly hindered the rapid permeation pathways for water molecules. Meanwhile, the enhanced hydrogen bonding interactions between Ag@ZIF-67 and the CMC matrix reduced the content of hydrophilic groups in the matrix, further weakening the binding affinity between water molecules and the matrix. Under these dual mechanisms, the WVTR of the composite film was significantly reduced to 0.39 [82]. Zhang et al. demonstrated that the addition of SiO_2_ to gellan gum/sodium carboxymethylcellulose/sodium alginate facilitated the formation of a dense cross-linked network structure that caused the oxygen permeability coefficient to decrease first and then increase. The dense network structure lengthens the tortuous pathway of oxygen diffusion, which leads to a decrease in the oxygen permeability coefficient of the film; however, excessive SiO_2_ incorporation leads to the accumulation of compounds in the film matrix, which impairs the structural integrity of the film, thereby reducing the film’s ability to block oxygen [81]. This phenomenon echoes the concentration-dependent behavior of nanomaterials in regulating the mechanical properties of films, which further confirms that the precise concentration regulation of nanomaterials is key to achieving a synergistic optimization of multiple properties in food preservation films, with the core lying in balancing the filling and cross-linking effects of nanomaterials and the structural homogeneity of the matrix.

In addition, nanoparticles can endow preservation films with excellent UV-shielding properties, further delaying the photooxidation of food lipids. Nguyen and Lee pointed out in their research that the introduction of only 1 wt% nano-TiO_2_ into a chitosan–gelatin/PVA/CNC-based composite film can reduce the average transmittance of UV-A (315–400 nm) and UV-B (290–315 nm) to 1.04% and 0.61%, respectively, corresponding to a shielding rate increase to approximately 99%. When the content of TiO_2_ is further increased to 5 wt%, the UPF value sharply increases from 1.43 of the pure film to 926.07, which is significantly better than the film without addition. Meanwhile, the transmittance in the visible light region (400–800 nm) only decreases by less than 10%, maintaining high transparency and thus satisfying consumers’ demand for visual inspection of food color [85]. This “ultraviolet–water oxygen” collaborative barrier strategy provides new ideas for the design of high-performance and biodegradable fresh-keeping films.

### 3.3. Thermal Properties

The thermal properties of food preservation films are one of the key indicators determining their practical application value, which is directly related to the films’ stability during processing, functional retention capacity under storage conditions, and durability against thermally induced degradation. Excellent thermal stability ensures that the films consistently exert their core fresh-keeping functions, such as barrier and antibacterial properties, while preventing structural damage or performance failure caused by thermal degradation. Existing studies have shown that the composite modification of nanomaterials and bioactive components has become an efficient approach to regulating the thermal properties of fresh-keeping films. Wang et al. showed that the addition of Daisy Essential Oil and nano-TiO_2_ to chitosan disrupted the intermolecular hydrogen bonds of chitosan, inducing the entanglement of chitosan molecular chains and forming a more stable composite network structure, thereby leading to a shift in the thermogravimetric analysis (TGA) curve toward higher temperatures (up to 403 °C) [84]. These findings indicate that the addition of nano-TiO_2_ and bioactive compounds effectively enhanced the thermal stability of the composite film, thereby improving its resistance to thermal degradation. Another study on soy protein nanofiber/chitosan composite films highlighted the synergistic enhancement of thermal properties by the “dual action mechanism” of nanoparticles and the ordering of the matrix structure. Wang et al. demonstrated via differential scanning calorimetry (DSC) tests that ZnONPs have a dual effect of “thermal insulation barrier and hydrogen bond cross-linking” in the soy protein nanofiber/chitosan film [86]. This effect elevates the peak denaturation temperature from 163.8 °C to 197.9 °C, significantly enhancing the thermal stability of the composite film. Meanwhile, after self-assembling into fibers, the protein changes from a free state to an ordered fiber network. The hydrogen-bond cross-linking becomes denser in this structured state, thereby further improving the thermal stability of the composite film. This result reveals that the enhancement of thermal performance by nanoparticles is not a single mechanism, but a synergistic effect of physical effects and chemical actions.

### 3.4. Antimicrobial and Antioxidant Properties

The synergistic interactions between nanomaterials and polymer matrices serve as a core modification strategy to overcome the limitations of single fresh-keeping functions and achieve the synergistic enhancement of the antimicrobial and antioxidant properties of food preservation films. By achieving complementary advantages through the superposition of mechanisms among different functional components, this strategy not only reinforces the biological protection efficacy of the films but also reconciles the demands of environmental friendliness, biocompatibility and practical applicability in food storage. The synergistic effect between inorganic nano-antimicrobial agents and plant-derived antimicrobial components is an effective strategy to address the shortcomings of single antimicrobial additives. As reported in the study by Motelica et al., the simultaneous introduction of “inorganic nano-bactericides (ZnONPs)” and a “plant essential oil controlled-release system (MCM-41@CEO)” into biodegradable hydroxyethyl cellulose preservation films not only can reduce the dosage of individual additives, but also can achieve long-acting antibacterial activity through a synergistic mechanism, providing a viable example for green antimicrobial packaging [93]. This self-assembled composite of multi-component broad-spectrum antibacterial nanomaterials can further broaden the antimicrobial spectrum of fresh-keeping films while accounting for biocompatibility, achieving the unity of antimicrobial performance and application safety. Xu et al. fabricated a self-assembled nanocomposite comprising three broad-spectrum antimicrobial materials (chitosan, graphene oxide and TiO_2_), which exhibited potent antimicrobial activity against *Aspergillus niger* and *Bacillus subtilis*, coupled with outstanding preservation performance [83]. Notably, the material showed no toxicity toward animal or plant cells, highlighting its biocompatibility. The synergy of multi-component antimicrobial materials is not a simple superposition of functions. Structural optimization achieved through self-assembly can also solve the problem of the biological toxicity of some antimicrobial materials, which is particularly suitable for edible packaging scenarios that are in direct contact with fresh food. The core value of the synergistic antibacterial modification of nanomaterials and polymer matrices must ultimately be reflected in its preservation efficiency in actual food storage. The study by La et al. verified both the in vitro antibacterial properties and the actual preservation effects of their developed coating. They incorporated ZnONPs into a chitosan/gum arabic edible coating, which exhibited potent antimicrobial activity against *S. aureus*, *E. coli* and *B. subtilis*. This coating effectively extended the shelf life of bananas from less than 13 days to over 17 days under conditions of 35 °C and 54% relative humidity while retaining nutrient contents during storage [94].

In addition to their antimicrobial properties, nanomaterials can also significantly enhance the antioxidant performance of films. As an important aspect of food preservation, antioxidant properties can inhibit the oxidative deterioration of food, thereby forming a dual preservation effect together with antibacterial properties. Li et al. evaluated malondialdehyde (MDA), superoxide dismutase (SOD) and peroxidase (POD) levels in preserved foods, and the results demonstrated that chitosan/SeNPs composite films exhibited superior antioxidant capacity compared to pure chitosan films. Specifically, SeNPs suppressed MDA accumulation, maintained SOD activity, and delayed the ripening process of fruits [5]. The detection of oxidation-related biochemical indicators also provides a quantitative basis for evaluating the antioxidant performance of fresh-keeping films, making the characterization of fresh-keeping effects more scientific.

### 3.5. Biodegradability

The soil burial method is a classic standardized method for evaluating the biodegradation performance and environmental risks of hydrocolloid-based composite food packaging films, as it can directly reflect the degradation dynamics of materials in natural soil [95]. Nanofillers represented by SiO_2_, Ag, Se, TiO_2_, ZnO and CDs exhibit distinct differential effects on the degradation performance and safety of composite films, owing to significant variations in their physicochemical properties and biological activities.

The role of SiO_2_ is dependent on its chemical form and interfacial interactions: mesoporous SiO_2_ (SBA-15), with its high specific surface area and excellent biocompatibility, does not form a degradation barrier in the chitosan/SBA-15-polyethyleneimine composite film. It can be completely degraded within 21 days, and the cell viability remains over 90% at a concentration of 1250 μg/mL, indicating that there are no significant toxic residues during the degradation process and thus high environmental safety [96]. In contrast, the inorganic SiO_2_ in the MNPs/Si/turmeric essential oil (TEO) system synergizes with TEO to inhibit microbial activity. After being buried in the soil for 6 weeks, the weight loss rate is only 65.48%, which is significantly lower than 90.72% of the pure chitosan film, exhibiting a degradation-delaying effect [97].

The potent antimicrobial properties of Ag and Se nanoparticles can target and inhibit the growth and reproduction of soil-degrading microorganisms, thus reducing the degradation efficiency of the composite film. Moreover, the inhibitory effect of AgNPs is positively correlated with their concentration [98]. However, this inhibitory effect is not absolute and is regulated by the intrinsic characteristics of the matrix material and structural design. For example, the degradation rate of the AgNPs@GT/EC (gelatin hydrogel/ethyl cellulose) double-layer film exceeds 90% in 19 days [99], and the degradation rate of the SC/Se 70 composite film reached 81% in 11 days [17], still retaining favorable degradation potential.

The TiO_2_-based composite film exhibits a phased degradation characteristic. The CMC-g-soy protein isolate/polyethyleneimine/TiO_2_ composite film maintains its complete structure in the first 30 days, initiates enzymatic degradation by soil microorganisms after 40 days, and achieves complete degradation in 64 days. Its efficient degradation is attributed to the synergistic effect of microbial enzymatic cleavage and ROS oxidation generated by UV-activated TiO_2_ [100].

The introduction of ZnO and CDs confers additional functionalities to composite films, such as antimicrobial activities and antioxidant properties, without impeding the degradation of the hydrogel matrix. The CSA18GZ3 nanocomposite film reached a weight loss rate of 56.68% within 30 days, while the DTB/ZnO/chitosan composite film exhibited significant fragmentation after 14 days—this degradation performance was notably superior to that of the PBAT film (which only developed slight microcracks) and the structurally intact PE film during the same period [101]. CDs synthesized through green methods from degradable plants, microorganisms, agricultural waste, etc., as a promising sustainable nanomaterial, generally do not hinder the degradation process [102]. Instead, they can indirectly affect the degradation rate by improving the film properties. For example, the degradation rates of konjac glucomannan (KGM)/Sodium Alginate and KGM/Sodium Alginate/nitrogen-doped carbon quantum dot (N-CQD) composite films are similar after 14 days of soil burial, and both achieve complete biodegradation [103].

Overall, under the premise of rational design, most nanofillers do not interfere with the soil degradation process of hydrocolloid-based composite food preservation films, and certain fillers can further enhance degradation efficiency through structural optimization or performance synergy. However, from the perspective of practical application scenarios, such materials have obvious functional conflicts: nanomaterials confer the composite films with excellent food preservation performance—they extend the shelf life of food by inhibiting the growth of pathogenic bacteria, but at the same time inhibit the activity of microorganisms involved in degradation in the soil, resulting in prolonged retention time of the materials in the natural environment, which in turn increases the risk of accumulation of nano-residues and ecotoxicity. Furthermore, there are still obvious deficiencies in current related research: most studies focus on the analysis of degradation performance in soil environments, but ignore the degradation behavior of materials in other real scenarios, such as aquatic environments and composting. Simultaneously, there is a lack of in-depth exploration on the residual laws and potential ecological impacts of nanomaterials in soil. These aspects require supplementation and refinement in future research.

### 3.6. Biosafety

The introduction of nanomaterials constitutes a core strategy for enhancing the functional properties of hydrocolloid-based composite food packaging films. Their migration behavior and safety thresholds are key factors governing the practical application potential of such materials. Under standard test conditions, the migration of different nanomaterials in composite films varies.

The European Union (EU) has not yet established specific migration limits for CDs, and existing research mainly focuses on verifying the biosafety of CDs. The experimental results showed that the hemolysis rate of the composite film containing CDs was less than 5%, indicating good blood compatibility. Additionally, its overall migration (OM) in food simulants was below the threshold of 10 mg/dm^2^ specified by EU regulations, meeting the general safety requirements for food contact materials [104]. SiO_2_ is authorized for unrestricted use in EU food contact materials regulations and has been assigned an ‘ADI (Acceptable Daily Intake) not specified’ by the European Food Safety Authority (EFSA), indicating its excellent safety profile comparable to the Generally Recognized as Safe (GRAS) status in the U.S. Food and Drug Administration (FDA) [105]. Relevant cytocompatibility experiments have confirmed that the chitosan/SBA-15-polyethyleneimine composite film maintained a viability of NIH 3T3 cells of above 90% even at a concentration as high as 1250 μg/mL, demonstrating outstanding biocompatibility [96]. For SeNPs, referring to the recommended standards of the U.S. National Academy of Sciences (NAS) and the Institute of Medicine (IOM), the recommended daily dietary selenium intake for adults is 55 μg, and the upper limit of the tolerable daily intake is 400 μg [5]. In vivo biosafety experiments further verified that the SC/Se 70 composite film exerted no adverse effects on healthy C57BL/6 mice. No significant differences were observed in body weight changes, pathological examination results, or liver and kidney function indicators between the treated and control group of mice, fully demonstrating the reliable biosafety of the film [17].

Compared with the aforementioned three types of nanomaterials, the migration level of TiO_2_ NPs is slightly higher. According to the requirements of the EU food contact plastics regulation 2020/1245, the specific migration limit (SML) for the migration of TiO_2_ in food contact plastics is 0.002 mg/kg of food or food simulant [105]. This SML is applicable to all food contact materials containing TiO_2_, which can effectively ensure human health and safety during their contact with food. When evaluating the cell viability of mouse fibroblasts (L929) in the presence of soy protein isolate/carboxymethyl chitosan/TiO_2_ nanotube arrays-3 films, the films demonstrated no obvious cytotoxicity and could also promote cell proliferation [18].

The migration levels of ZnONPs and AgNPs are relatively elevated. AgNPs are often incorporated into chitosan-based composite coatings to enhance antimicrobial properties and extend the shelf life of food. Nevertheless, excessive silver migration can present a potential threat to human health, attributed to the concentration-dependent toxicity of silver. Films prepared by introducing natural Kaolin clay and Ficus carica-mediated AgNPs into chitosan had a silver migration amount of 7–8 μg/kg·dm^2^ in water on the 1st day, which increased to 19–21 μg/kg·dm^2^ on the 5th day [95]. In a C4E3Ag3 film fabricated by adding AgNPs synthesized with dragon fruit stem extract into a chitosan-based film, the silver migration rate was 8.95 ± 0.25 μg/kg·dm^2^ on the 1st day of immersion and increased to 21.96 ± 0.43 μg/kg·dm^2^ on the 5th day [106]. The silver migration amounts of both samples were below the silver migration limit for food packaging established by the EFSA (0.05 mg/kg food) [107] and the daily allowable intake of silver for humans set by the European Chemicals Agency (ECHA) (0.9 μg Ag/kg body weight/day) [108]. Furthermore, the contact area between the film and food in practical applications is much smaller than that in the full immersion test conditions, further reducing the risk of silver migration [106]. Therefore, it can serve as a safe short-term food packaging material. The EU plastics regulation EU10/2011 sets the SML of ZnO at 25 mg/kg (expressed as zinc), while the U.S. Food and Drug Administration (FDA) assesses its safety based on the tolerable upper limit of 40 mg/person/day [109]. Numerous studies have demonstrated that the migration level of ZnONPs from packaging materials to food is extremely low and poses no threat to human health [110]. However, ZnONPs with a concentration of 50 μg/mL can exhibit cytotoxicity and genotoxicity to nasal mucosal cells [111].

## 4. Research Progress on Hydrocolloid Matrix and Nanomaterial Composite Films

**Figure 3:**
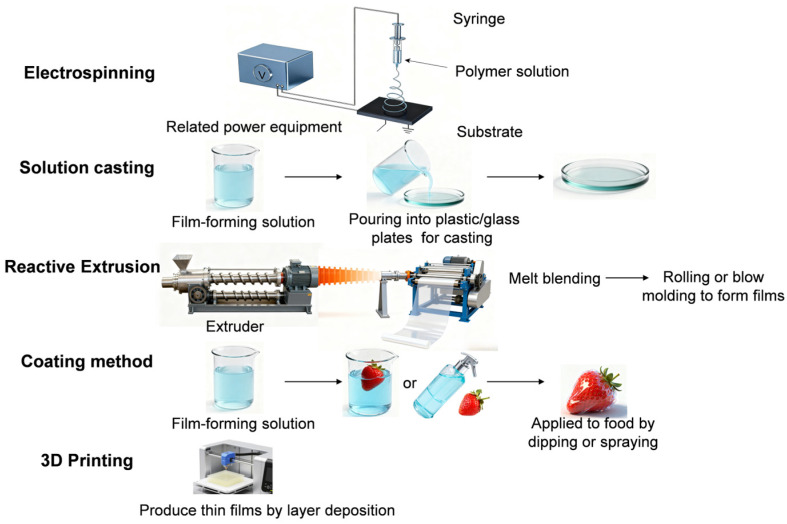
Schematic illustration of common preparation methods for composite preservation films: electrospinning, solution casting, blending modification, and reactive extrusion.

The fabrication methods of hydrocolloid-based composite food preservation films exert a direct impact on their microstructural characteristics and functional properties. Common fabrication techniques include electrospinning, solution casting, reactive extrusion, the coating method, and 3D Printing (Figure 3).

### 4.1. Electrospinning

Electrospinning, as an advanced nanofilm-forming technology, enables the fabrication of nanofibrous films with a high specific surface area and porous structure by virtue of its characteristic of electrostatic force-driven polymer stretching and fiber formation [112]. Its unique fim structure and tunable fiber morphology render it a promising technical approach for the preparation of high-performance food preservation packaging films. Studies by Dodero et al. on alginate-based electrospinning films have revealed the crucial role of the combination of polymer matrix structure regulation and post-treatment processes in film stability [113]. The molecular weight and M/G ratio of alginate are core parameters that determine its film-forming ability and structural stability. In the study, a highly stable alginate nanocomposite film with the electrospinning-specific nanofibrous structure fully retained was successfully fabricated by the preferential selection of alginate with a low molecular weight and high M/G ratio or a medium molecular weight and low M/G ratio for composite formation with ZnONPs, coupled with post-treatment strategies of hot ethanol washing and strontium ion cross-linking. Banitaba et al. identified the regulatory mechanism of ratio regulation between functional fillers and polymer components on fiber morphology and the mechanical properties of films in their investigation on a PVA/pectin-ZnO electrospinning system [114]. This also confirms that the ratio compatibility between functional nanofillers and the polymer matrix is a crucial element in regulating the performance of electrospinning. As the proportion of pectin and ZnO increased synchronously, the fibers became finer, and the tensile strength was enhanced. He et al. used electrospinning technology to prepare GA-PVA/chitosan/tannic acid@ZnO nanofiber films. Their mechanical strength increased from 3 MPa to 18 MPa, and the heat resistance temperature rose from 225 °C to 310 °C. Chitosan, tannic acid and ZnO formed a synergistic antimicrobial effect, making the material’s bacterial inhibition rate exceed 99.9%, and cytotoxicity tests confirmed that it was non-toxic. After application, the film could effectively extend the freshness period of strawberries to 6 days, providing a new path for the development of green multifunctional food packaging [115]. The porous nanofibrous structure of electrospun films not only provides uniform loading sites for functional components and reinforces the synergistic effects of multi-component systems, but also adapts to the respiratory characteristics of fruits and vegetables by balancing gas exchange and moisture retention during the preservation process, thus enabling the effective conversion of laboratory-scale performance into practical food preservation efficacy. Electrospinning technology has emerged as a prominent technical option for the fabrication of hydrocolloid-based composite food packaging films, owing to its inherent process merits—such as reduced polymer consumption, tunable fiber diameter, high porosity and designable multilayer structures [116,117]. However, electrospinning technology exhibits notable limitations in the fabrication of functional nanocomposite films for food packaging. Firstly, the equipment required for this technology entails high procurement and operational costs, thus limiting its application primarily to the high-value-added medical sector. Secondly, the control accuracy requirements for key parameters such as voltage and solution concentration are stringent, which restricts the industrialization promotion process.

### 4.2. Solution Casting

Solution casting, as a widely used traditional technology for the fabrication of food packaging composite films, acts as a core method for the rapid screening of film-forming formulations in the laboratory [118]. Its core advantages lie in a simple operational process, low equipment threshold and high cost-effectiveness, which affords an efficient approach for the initial development of green food preservation films. Lian et al. achieved the simultaneous enhancement of a film’s microstructure and functional properties by introducing TiO_2_ NPs into the casting film-forming solution and combining this with a high hydrostatic pressure (HHP) post-treatment [119]. The physical filling effect of TiO_2_ NPs optimized the compactness of the film, thereby improving its water/gas barrier properties; their intrinsic antimicrobial activity produced a synergistic effect with the natural bacteriostatic activity of chitosan while also enhancing the film’s mechanical strength. In contrast, HHP post-treatment further facilitated the interfacial interactions between polymer molecular chains and nanoparticles, reduced internal defects within the system, and thus rendered the modification effect more stable. Although this method is simple, economical and efficient, with the film-forming process requiring only three steps—dispersion, casting, and drying—and no need for precision equipment, it is limited to small-scale laboratory production [120]. First and foremost, it is difficult to precisely control the film thickness uniformity during the casting process, and the drying of films in large-scale casting poses challenges in addressing issues such as uneven heat transfer and excessively long drying time. Furthermore, in large-scale production, the scaling-up of the solution system tends to induce the agglomeration of nanoparticles, which undermines the homogeneity of the film’s microstructure and thus impairs the stability of its modification effect. Collectively, these issues render solution casting unable to meet the core requirements of the food packaging industry for product standardization and mass supply.

### 4.3. Reactive Extrusion

Reactive extrusion, as a hybrid technology integrating chemical reactions with extrusion processing, has a core advantage in the integration of modification and forming processes for biodegradable polymers [121]. Herniou et al. fabricated cellulose acetate/corn thermoplastic starch composite blend films via reactive extrusion [122]. They found that films incorporating a chromium octoate catalyst exhibited shape memory behavior and enhanced mechanical performance, thus demonstrating promising potential for the development of shape memory food packaging materials. More importantly, reactive extrusion achieves continuous and efficient industrial-scale production via the integrated processes of calendering and blow molding, thereby completely overcoming the limitations of small-scale production associated with the solution casting method [123]. Its production efficiency and cost control capability are fully aligned with the mass supply demands of the food packaging industry, laying a solid foundation for the industrialization of green packaging materials.

### 4.4. Coating Method

The coating method’s core advantage lies in the formation of a semi-permeable film on the food surface via a composite solution of hydrocolloid matrices and nanomaterials through simple dipping or spraying approaches. This film not only acts as a physical protective barrier, adapting to the postharvest respiratory metabolism of fruits and vegetables by regulating the gas exchange rate, but also inhibits microbial penetration, thus retarding food spoilage from the dual dimensions of physical barrier and biological protection [124]. Xu et al. prepared a coating by a one-step self-assembly of tea polyphenols (TP) and Zn^2+^ [125]. The coating has strong adhesion and can adhere to the surface of fruits. One of the key pain points of postharvest fresh-keeping coatings for fruits and vegetables is their tendency to detach due to transportation friction and moisture evaporation, whereas this self-assembled coating forms a network structure with an appropriate cross-linking degree via a coordinated interaction between the phenolic hydroxyl groups in TP molecules and Zn^2+^ ions. This endows the coating with the ability to firmly adhere to fruit surfaces, thereby providing a structural foundation for the sustained exertion of subsequent fresh-keeping functions. In terms of its fresh-keeping effect, the coating has a significant effect on extending the shelf life of strawberries and bananas: the shelf life of strawberries is extended by 6 days at room temperature and 25 days under refrigeration, while the shelf life of bananas is extended by 8 days, with effective inhibition of browning and maintaining hardness and nutrition. This method features facile operation and feasibility at ambient temperature, thus being applicable to small-scale production. However, coatings prepared via this approach exhibit a pronounced variation in affinity for the surfaces of different fruit cultivars, and their fresh-keeping performance displays significant fluctuations with changes in environmental conditions, thereby impeding their scalability for large-scale industrial manufacturing [126].

### 4.5. 3D Printing

3D printing is an emerging technology that produces thin films layer by layer, featuring high production efficiency and the capability to freely design the shape, size, porosity, and other characteristics of thin film labels [127]. Chen et al. fabricated a double-layer active packaging film composed of gelatin/PVA/banana peel carbon dots in its outer layer and corn starch/PVA/cinnamon essential oil in its inner layer through 3D printing technology, which has various functions such as antioxidant activity, antimicrobial effects, UV-shielding capacity and the controlled release of essential oils [87]. Experiments demonstrated that the film can significantly enhance the retention rate of cinnamon essential oil during drying, and enables its sustained release during storage, effectively prolonging the fresh-keeping period of mango while improving the flavor stability of spicy essential oil microcapsules. 3D printing technology is still in the functional exploration phase for the preparation of preservation films and faces problems such as strong dependence on specialized printing equipment and relatively thick film thicknesses. However, due to its prominent advantages, it is regarded as a promising technology for the fabrication of preservation films.

Different fabrication methods exhibit distinct characteristics regarding the performance, cost, and application scenarios for composite preservation films. Laboratory research predominantly employs solution casting and coating methods, while electrospinning and reactive extrusion are pivotal for industrial-scale production. The solution casting method, with its mild fabrication conditions, can form a film matrix with a dense structure and uniform thickness, which is suitable for small-scale formulation optimization and preliminary performance exploration. The coating method, by contrast, uniformly applies the composite fresh-keeping slurry onto the substrate surface and forms a functional coating through drying and curing. It is commonly applied for the surface functionalization treatment of modified traditional packaging materials. For industrial-scale production, the electrospinning method and reactive extrusion are particularly critical. The electrospinning method stretches polymer solutions or melts into nanofibers under a high-voltage electric field, enabling the construction of a nanofiber network with a high specific surface area and high porosity. The reactive extrusion method, on the other hand, completes the integration process of polymer melting, mixing, reaction, and molding in an extruder. It has high production efficiency and excellent process continuity, which can meet the demands of large-scale industrial production. Additionally, as an emerging fabrication method, 3D printing technology can precisely control the microstructure and functional area distribution of the film based on the principle of layer-by-layer stacking, providing a novel solution to specialized food packaging requirements.

Future efforts should focus on developing low-temperature, energy-efficient green processes and overcoming key challenges such as nanomaterial dispersion and polysaccharide thermal stability. In the field of food packaging, the development of high-performance composite films is crucial for extending shelf life, preserving quality and reducing food waste [128]. With advancing technology and growing demand for eco-friendly and safe materials, researchers are optimizing film properties by adjusting hydrocolloid-to-nanomaterial ratios and incorporating plasticizers [129].

### 4.6. Nanomaterial Proportion Control

The added amount of nanomaterials is a key parameter in regulating the comprehensive performance of hydrocolloid-based composite food packaging films. Appropriately increasing the content of nanomaterials can significantly improve the core performance of the films through interface interaction and structural optimization. For example, Ruchir et al. improved chitosan film performance by incorporating varying concentrations of ZnO nanoparticles. Higher ZnO levels slightly improved thermal stability and substantially boosted mechanical durability [130]. In addition to mechanical properties, nanomaterials can also regulate the optical properties of films, and they perform particularly well in the field of UV blocking. Jo et al. observed that Ag/LDPE and Ag/PP nanocomposite films exhibited yellowing and enhanced UV absorbance in the 400–500 nm range as the AgNPs concentration increased. However, excessive AgNPs may increase brittleness and processing complexity while posing potential safety concerns [131]. Indumathi et al. identified 5% ZnO (*w*/*w*) as the optimal ratio for chitosan/cellulose acetate phthalate films, which maximized TS and hardness while maintaining favorable flexibility [132]. Beyond this threshold, film flexibility decreased significantly, whereas brittleness increased.

### 4.7. Plasticizer Integration

Plasticizers (e.g., glycerol, ethylene glycol, sorbitol) penetrate polymer chains, reducing intermolecular forces to improve flexibility and gas permeability [133,134]. For instance, Zdanowicz et al. used the three-component deep eutectic solvent of choline citrate-urea-glycerol (CCit:U:G) as a “crosslinking plasticizer” [135]. The study demonstrated that during the film-forming process, citrate ions react with starch hydroxyl groups through an esterification reaction to form a covalent network, anchoring the DES molecules within the matrix and significantly inhibiting their migration in the film. However, it is worth noting that when the additional amount of nanofillers in the system is too high, it will significantly inhibit the above cross-linking reaction. Just as the citrate ions in CCit are easily adsorbed on the surface of montmorillonite, resulting in a decrease in the effective sites for esterification cross-linking with starch hydroxyl groups, ultimately weakening the construction efficiency of the cross-linking network [136], Jha demonstrated that incorporating glycerol or sorbitol into corn starch–chitosan films enhanced their mechanical properties [137]. Similarly, González-Torres et al. blended oxidized potato starch with sorbitol and glycerol, achieving films with improved TS and elasticity [138].

## 5. Interaction Mechanisms Between Nanomaterials and Hydrocolloid Matrix

In composite preservation films, the binding mechanisms between nanomaterials and the hydrocolloid matrix are complex, primarily achieved through multiple interactions to form tight combinations that enhance film performance.

### 5.1. Electrostatic Interactions

Electrostatic interactions constitute a key binding mechanism between nanomaterials and hydrocolloid matrices. For instance, the carboxyl groups (-COOH) on the carboxymethyl cellulose molecular chains dissociate into negatively charged -COO^−^ in aqueous solutions, enabling them to modulate the formation of metal nanoparticles through a dual effect: on the one hand, acting as a stabilizer to provide electrostatic repulsion, and on the other hand, exerting a reducing function through hydroxyl groups (-OH). In the synthesis of AgNPs, the negatively charged CMC chains directionally adsorb positively charged Ag^+^ ions through electrostatic attraction to form stable ion-polymer complexes. Subsequently, the hydroxyl groups of CMC reduce Ag^+^ to AgNPs, while electrostatic encapsulation effectively inhibits nanoparticle aggregation [139]. In another study, Ding et al. incorporated a novel nanofiller—poly (tannic acid)-TiO_2_ (TP-NPs)—into chitosan films, fabricating multifunctional chitosan films with visible-light-responsive photocatalytic properties [140]. The electrostatic interactions and robust hydrogen bonding between TP-NPs and chitosan chains significantly enhanced the mechanical properties of the films.

### 5.2. Hydrogen Bonding

Hydrogen bonding constitutes another prominent interaction mechanism. Surface functional groups of nanomaterials can form hydrogen bonds with hydroxyl (-OH) or amino (-NH_2_) groups in hydrocolloid matrices. The redshift phenomenon of group vibration peaks, characterized by Fourier transform infrared spectroscopy (FTIR), can serve as robust evidence for the occurrence of hydrogen bonding interactions. Xu et al. have corroborated this correlation through relevant research [141]: upon incorporating TP-stabilized ZnONPs (TZNP) into a chitosan matrix, the electron-rich -OH and -NH_2_ groups on chitosan molecular chains act as hydrogen bond donors. In contrast, TP-coated or surface-hydroxylated ZnO exhibits dual characteristics as both a proton donor and acceptor. These complementary properties facilitate the formation of intermolecular hydrogen bonds between TZNP and chitosan, which ultimately induces a redshift in the vibration absorption peaks corresponding to chitosan’s -OH and -NH groups. For example, in TiO_2_ NPs-nisin-modified chitosan composite preservation films, shifts in the characteristic amide I band (e.g., from 1650 cm^−1^ to 1638 cm^−1^) indicate the formation of hydrogen bonds between TiO_2_, nisin and chitosan [142]. In chitosan–andrographolide extract–SeNPs composite films, SeNPs form hydrogen bonds with the ascorbyl palmitate and polyphenolic compounds in chitosan, ensuring their stable integration into the film matrix [143]. Studies on CuO-CHNF (cellulose nanofiber) and CuO-BCNF (bacterial cellulose nanofiber) films have further revealed that CuO NPs are immobilized through dual mechanisms: (1) hydrogen bonding between surface hydroxyl groups of nanofibers and CuO, and (2) electrostatic interactions between Cu^2+^ ions and electron-rich oxygen atoms in polar hydroxyl/ether groups of BCNF/CHNF [144].

### 5.3. Coordination Interactions

Beyond the aforementioned interaction mechanisms, coordination interactions between metal atoms in nanomaterials and specific functional groups of hydrocolloid matrices further stabilize their binding and endow films with antimicrobial properties. For example, in TiO_2_/chitosan nanocomposite coatings, coordination bonds are formed between Ti atoms and amino groups (-NH_2_) of chitosan, reinforcing functional performance [145]. Ti^4+^ is classified as a “hard acid” (characterized by a small ionic radius, high charge density, and low polarizability of the outer electron cloud), while the N atom in the amino group (-NH_2_) acts as a “hard base” (characterized by the facile donation of lone electron pairs and low electron cloud polarizability). In line with the principle of “hard acids preferentially bind to hard bases” proposed by the Hard and Soft Acids and Bases (HSAB) theory, these two species readily form stable coordination bonds [146]. Collectively, these synergistic interaction mechanisms enable the formation of stable nanostructures within composite films, thereby optimizing their comprehensive preservation performance.

## 6. Prospects and Challenges of Composite Preservation Films

**Figure 4:**
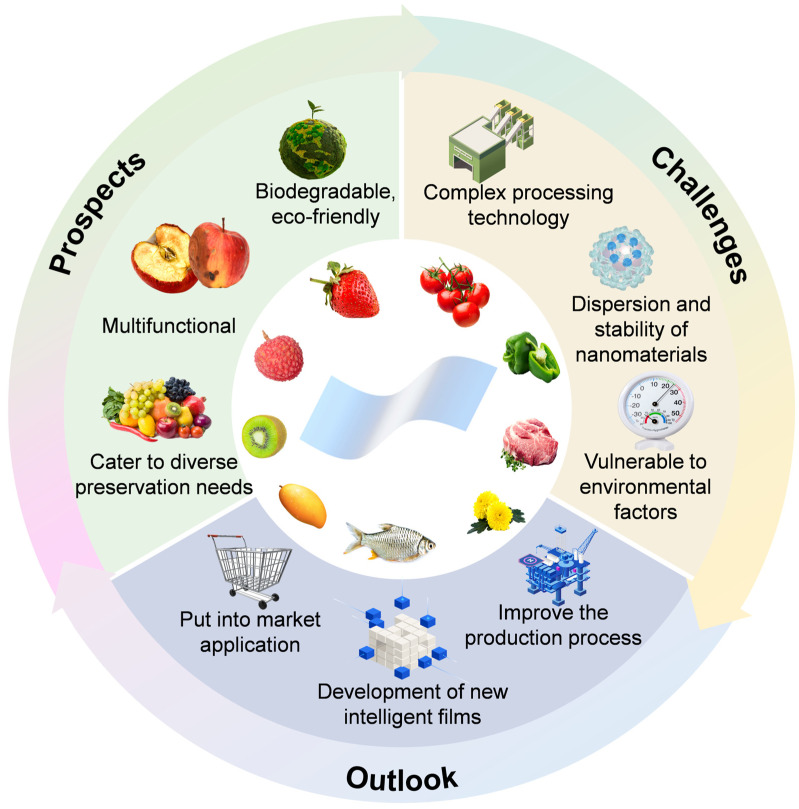
Application prospects, challenges, and outlook of hydrocolloid-nanomaterial composite films.

In the future, composite films with biodegradability, biosafety, and multifunctionality will accurately respond to diverse fresh-keeping requirements and emerge as core materials in the food preservation industry. Despite their inherent advantages, composite films face significant challenges. First, their production involves complex multi-material processes (e.g., blending, coating, lamination), presenting technical obstacles to scalable production [147]. Poor dispersion and stability of nanomaterials may lead to inconsistent film performance. Safety concerns persist: certain nanoparticles may pose chronic toxicity risks, such as DNA damage, cytotoxicity, or carcinogenicity [148], while migration risks and long-term biosafety remain understudied [149]. Rigorous toxicological evaluations are essential to safeguard food safety. Additionally, regional fluctuations in temperature and humidity may affect preservation efficacy, which necessitates further research. Although composite films with diverse functionalities, such as temperature-sensitive [150], humidity-responsive [151], and extended preservation of fruits and vegetables under high-humidity conditions [152], have been developed, their large-scale production and practical application still need to be verified. The global food packaging market value exceeded $450 billion in 2024, and it is expected to grow at a compound annual growth rate of 5.6% from 2025 to 2034, which provides a huge entry point for green alternative materials [153]. Currently, most related studies remain in the developmental phase, requiring optimization of preparation processes, cost reduction and enhanced scalability. Comprehensive evaluations of safety and environmental impact are critical to ensure reliability and sustainability (Figure 4).

## 7. Literature Review: The PRISMA Method

**Figure 5:**
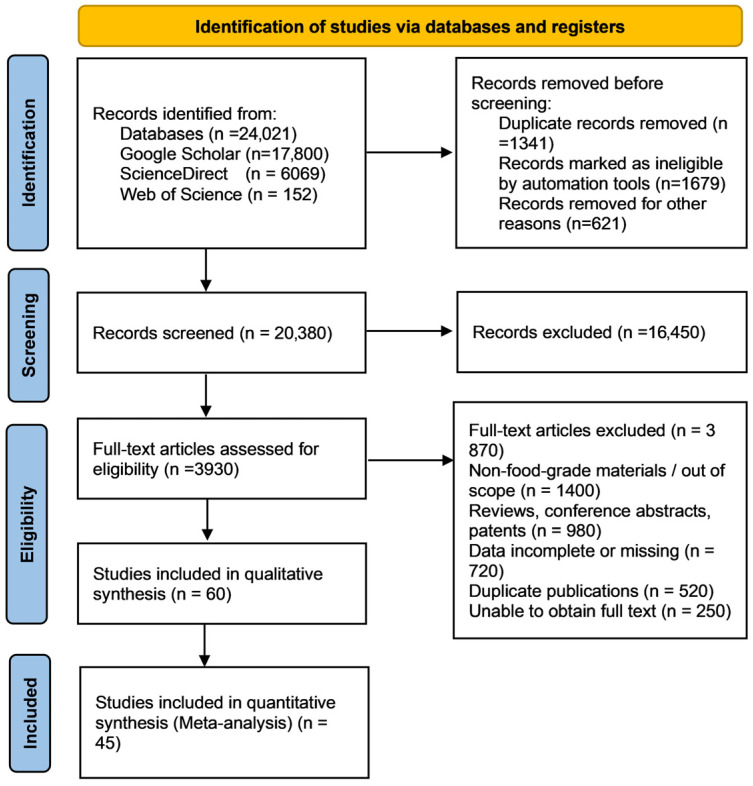
PRISMA flow diagram.

To systematically evaluate the application effect of the hydrocolloid–nanomaterial composite preservation system in fresh food, this study followed the PRISMA 2020 guidelines and constructed search formulas in the three databases of ScienceDirect, Google Scholar and Web of Science: (hydrocolloid OR “bio-based gel” OR “edible coating”) AND (nano OR “nanocomposite” OR “nanoparticle”) AND (preservation OR “shelf-life” OR “food preservation”). The time span was from 1 January 2015 to 31 December 2025, and the language was limited to English. A total of 24,021 articles were obtained in the initial search, including 17,800 from Google Scholar, 6069 from ScienceDirect, and 152 from Web of Science. After removing 3641 duplicates automatically and manually by EndNote, 20,380 articles entered the preliminary screening of titles/abstracts. According to the PICOS framework, 16,450 articles were excluded, and the remaining 3930 articles were accepted for full-text eligibility assessment. Inclusion criteria: (1) The research objects were fresh or minimally processed foods; (2) the intervention measures were coatings, films, or active packaging made of hydrocolloid matrices (≤100 μm) loaded with food-grade nanomaterials (≤100 nm); (3) untreated, pure hydrocolloid, or traditional packaging controls were used; (4) one of the microbial, physical–chemical, or sensory indicators was reported and the mean ± SD was given; (5) experimental peer-reviewed papers. Exclusion criteria: Non-food-grade materials, reviews, conference abstracts, patents, missing data, duplicate publications, unobtainable full texts, or those unrelated to preservation. After double independent reviews and cross-checks, a total of 60 articles were included in the qualitative synthesis, and 45 of them with complete data entered the quantitative meta-analysis. The quality was evaluated using the JBI experimental research checklist, with an average score of 7.8 ± 0.9, and studies with a low risk of bias accounted for 78%. This process, for the first time, provided a PRISMA evidence map that complies with international norms for the theme of “hydrocolloid–nanomaterial preservation”, laying an evidence-based foundation for subsequent safety, scale-up, and regulatory decisions (Figure 5).

## 8. Conclusions

As a cutting-edge direction in food packaging, hydrocolloid-nanomaterial composite films leverage the biodegradability and biocompatibility of polysaccharides while integrating functional nanomaterials to deliver exceptional antimicrobial, antioxidant and barrier properties. These films offer innovative solutions to the environmental pollution and performance limitations of traditional plastics. They not only reduce ecological burdens but also enhance food preservation, meeting modern demands for efficient, intelligent and sustainable packaging. However, the current composite films still face bottlenecks, such as challenges in industrial-scale production, the unclear nanomaterial safety profiles and migration behaviors of nanoparticles, and the challenge of balancing multiple functions. The solution casting method is the most widely reported approach for film preparation in the existing literature. Therefore, the development of new, innovative, and low-energy-consuming preparation methods will also become a key research goal in this field for the future. To advance large-scale applications, research should prioritize developing low-energy, eco-friendly processes to minimize thermal degradation and nanoparticle aggregation; designing multi-functional films with pH-, temperature-, or gas-sensing capabilities for diverse market needs; establishing standardized protocols for nanomaterial toxicity and migration risk assessments; and tailoring material ratios to balance mechanical strength, flexibility and barrier properties. By addressing these challenges, hydrocolloid–nanomaterial composites can revolutionize food packaging, paving the way for safer, smarter, and more sustainable solutions. This review is mainly based on the induction of existing literature. There is still a lack of discussion on the migration amounts of nanomaterials, long-term storage stability, and the regulatory frameworks of nanomaterials. The conclusions still remain at the laboratory verification level and require further analysis and verification through subsequent experiments.

## Figures and Tables

**Table 1:** Application of hydrocolloid matrix in preservation films.

Hydrocolloid Matrix	Other Materials	Modification	Key Findings	References
Chitosan	Bacterial cellulose, Curcumin	Enhanced hydrophobicity, oxygen barrier property, mechanical strength and antioxidant activity.	Chitosan was grafted onto bacterial cellulose via Schiff base reactions, producing a biodegradable film with superior performance.	[26]
Chitosan	Aluminum chloride, Cellulose	Reduced tensile strength and Young’s modulus but increased elongation at break and antimicrobial activity.	Innovative incorporation of AlCl_3_ improved the hydrophilicity and mechanical properties of the composite film.	[27]
Gelatin	Resveratrol, Carboxymethyl cellulose	Reduced water vapor permeability (WVP) and enhanced thermal stability, antioxidant activity and hydrophobicity.	Resveratrol acted as a hydrophobic agent, achieving 85% DPPH and ABTS radical scavenging efficiency. The film effectively inhibited soybean oil oxidation.	[32]
Gelatin	Zein nanoparticles, Quercetin	Improved tensile strength, water/oxygen barrier capacity, hydrophobicity, UV-shielding efficiency, antioxidant activity and antimicrobial performance.	The composite film reduced WVP by 78.4% compared to pure gelatin film and extended strawberry shelf life to 8 days.	[33]
Sodium Alginate	Carboxymethyl chitosan, Citric acid	Excellent mechanical properties, low moisture permeability, antimicrobial performance and recyclability.	Films were prepared via electrostatic interactions between oppositely charged polysaccharides. The film retained excellent preservation performance even after regeneration.	[38]
Sodium Alginate	Chitosan, TiO_2_	Enhanced mechanical strength, barrier properties, antimicrobial activity and color stability.	Anthocyanins improved antimicrobial and antioxidant properties. TiO_2_ nanoparticles stabilized the bilayer film color. The bilayer film extended pork shelf life and enabled real-time freshness monitoring.	[39]
Starch	MgO nanoparticles, Tea polyphenol	Improved tensile strength, UV-shielding efficiency, hydrophobicity and thermal stability; reduced water content, water solubility and WVP.	Synergistic incorporation of tea polyphenol and MgO nanoparticles enhanced film performance. The film degraded completely within ~20 days under simulated environmental conditions, demonstrating biodegradability.	[48]
CMS	ZnONPs, Chitosan	The tensile strength and water vapor barrier property were increased by 147% and 73%, respectively, compared with the pure chitosan film. The light transmittance was close to 89%, the antimicrobial duration exceeded 202 h and strawberries remained fresh within 5 days.	This film is sensitive to humidity and can monitor the humidity of the fruit storage environment in real time.	[51]
CMC	TiO_2_, Copper	Enhanced water vapor barrier and mechanical properties. Cu-doped TiO_2_ exhibited higher visible-light photocatalytic activity against foodborne pathogens.	Cu-TiO_2_-doped films showed significant antimicrobial activity under visible light.	[52]
Pectin	Citrus flavonoids, Soy protein	Improved mechanical strength, thermal stability, gas barrier properties, antioxidant activity and antimicrobial performance.	Pectin and flavonoids were extracted from citrus peels and combined with soy protein isolate to prepare films via solution casting	[53]
Pectin	Propolis extract, Zein, Fe^3+^	Reduced water content (~46%) and WVP (~19%); doubled tensile strength and antioxidant activity; improved antimicrobial performance.	Dual crosslinking with zein nanoparticles and Fe^3+^ enabled controlled release of propolis extract (PPE), prolonging its preservation effect.	[54]
Hyaluronic Acid	Cinnamaldehyde, HP-β-CD	Enhanced tensile strength, water vapor barrier, antimicrobial activity, antioxidant activity and UV-shielding efficiency.	Cinnamaldehyde HP-β-CD inclusion complex enabled sustained release kinetics, extending preservation efficacy.	[55]
Hyaluronic Acid	Chitosan, Gelatin	Reduced weight loss, slower pH decline, delayed TA reduction, and stabilized TSS levels.	Higher hyaluronic acid content correlated with stronger antioxidant activity (via total phenolics, ascorbic acid and DPPH assays).	[56]

**Table 2:** Roles of nanomaterials in preservation films.

Nanomaterial	Concentration	Film-Forming Substrate	Function and Effect					Reference
			Mechanical Properties	Barrier Properties	Thermal Properties	Antimicrobial and Antioxidant Properties	Preservation Efficacy	
SiO_2_	1%	Gellan gum, Sodium carboxymethylcellulose, Sodium alginate	TS increased by 33.6%.	WVP minimized to 2.32 × 10^−11^ g·m^−1^·s^−1^·Pa^−1^, oxygen permeability (OP) coefficient decreased by 57.1%.	Maximum decomposition temperature increased from 227 °C to 230 °C.	With the increase in added SiO_2_, the bacteriostatic zone increased significantly, and the free radical scavenging rate of DPPH increased.	The control group began to decompose on day 8, and the experimental group remained commercial on days 10–12.	[81]
Se	2%	Chitosan	TS increased from 19.7 MPa to 24.4 MPa, and the EB rose from 12.5% to 14.7%.	The WVP value of CTS/Se film decreased by about 6% compared with that of CTS film.	The thermal stability of the CTS/Se film was observed to be slightly superior to that of the CTS film.	After 10 days of storage, there were significant differences in the surface colony counts of tomatoes among different treatment groups: the surface colonies of toma-toes in the CTS/Se coating group were almost eliminated, with only 1 remaining; a small number of colony spots appeared on the surface of tomatoes in the CTS coating group; while a large number of colonies grew on the surface of tomatoes in the control group.	After 20 days of storage, the epidermal wrinkles of the control group were more severe, while those of the CTS/Se coating group were slight and the color was better maintained.	[5]
Se	SC/Se 70	Quaternized Chitosan, Epigallocatechin Gallate (EGCG)	Both TS and EB increased at high concentration.	WVP reduced by about 6%, water contact angle close to 90°, UV barrier property enhanced.	Weight loss reduced to 66.57%.	The DPPH scavenging rate and antimicrobial properties of the film increase with the increase in concentration of SeNPs.	After 120 h of storage, decay rates of lychees were 53% (control), 100% (PE film), and slight (SC film). After 360 h, the control group and SC film group generally became moldy, and only the SC/Se 70 film group remained fresh. The decay rate and weight loss rate are significantly reduced, and the hardness, total soluble solids (TSS), titratable acidity (TA), and flavor are better maintained.	[17]
Ag	-	CMC, zeolitic imidazolate framework-67	The EB increased by approximately 16%, the TS increased by approximately 31%, and the film thickened slightly.	WVP decreased by about 5%, OP decreased by 12.4%, and carbon dioxide permeability (CDP) decreased by 25.35%.	The final decomposition temperature is increased by about 5 °C, and the thermal decomposition is delayed.	Lethality rate of *S. aureus* treated with Ag@ZIF-67 reached 99.98% (20 μg/mL, 6 h).	Fruit surface remained free of mildew and plump after 6 days of storage at 25 °C (compared with the control group).	[82]
TiO_2_	NPs2 (GO:Chitosan:TiO_2_ = 1:20:4)	Graphene oxide, Chitosan	-	-	-	NPs2 nanomaterial exhibited significant antimicrobial effects against biofilm formation of *B. subtilis* and *A. niger*.	Effectively delays loss of moisture in fruits and vegetables, inhibits polyphenol oxidase (PPO) activity, and enhances antioxidant enzyme activity, including SOD activity.	[83]
TiO_2_	-	Chitosan, Daisy Essential Oil	TS increased by 6-fold compared with pure chitosan film, and the EB decreased by about 41.8%.	Chitosan-TiO_2_-DEO (CSTD) films have the lowest WVP and the highest CDP.	The addition of nano-TiO_2_ and essential oils shifted the TGA curve to higher temperatures, reaching 403 °C.	The CSTD film demonstrated the strongest DPPH radical scavenging ability, achieving a clearance rate of 89.65%. The CSTD film exhibited the highest antimicrobial activity, with inhibition zones of 25.842 ± 0.731 mm for *E. coli* and 26.631 ± 0.410 mm for *S. aureus*.	The antioxidant properties of DEO and nano-TiO_2_ enhanced the internal antioxidant capacity of fruits, maintained high levels of total phenolic content (TPC) and total flavonoid content (TFC), thereby improving the storage quality of kiwifruit and extending its shelf life to 10 days.	[84]
TiO_2_	5 wt% TiO_2_ in the PVA/CNC matrix with 5 wt% of CNCS	PVA, cellulose nanocrystals (CNC)	In the thin film with a low TiO_2_ content (1 wt%), TiO_2_ and CNC synergistically cross-linked, increasing TS and Young’s modulus by approximately 55% and 45%, respectively. However, excess TiO_2_ caused decreases due to agglomeration; EB decreased with increasing TiO_2_ content.	10 wt% TiO_2_ reduces WVP by 46.8%, decreases the UVA/UVB transmittance to less than 1%, and boosts the ultraviolet protection factor (UPF) to 952.37.	-	The PVA/CNC/TiO_2_ 5% and PVA/CNC/TiO_2_ 10% films exhibited excellent antimicrobial activity. At the end of the experiment (21 days), the microorganisms only covered 49.1% and 34.1% of the milk surface, which was attributed to the continuous generation of reactive oxygen species (ROS) by TiO_2_ under ultraviolet excitation.	At room temperature (23–25 °C), the weight loss of garlic decreased from 32.6% of the unpacked garlic to 11.2% in 14 days. Fresh garlic packaged in PVA/CNC/TiO_2_ 5% and 10% bags almost maintained its original shape and color within 14 days without obvious spoilage.	[85]
ZnO	0.5%	Pectin	The addition of 0.5% ZnO slightly decreased the TS of the pectin film to 2.17 MPa and slightly increased the EB to 16.28%.	WVP decreased to 8.85 g·mm/m^2^·d·kPa, significantly lower than that of the control (13.22 g·mm/m^2^·d·kPa).	-	The pectin film in the control group itself has antimicrobial activity, and the addition of ZnO does not significantly enhance it.	The edible coating incorporated with 0.5% ZnONPs based on pequi pectin can control the weight loss rate of mangoes below 10% within 12 days, inhibit respiration and ethylene synthesis, delay yellowing and softening, and improve shelf quality.	[19]
ZnO	-	Chitosan, Soy protein nanofiber	TS increased by 4-fold.	The addition of ZnO decreased the swelling rate and water solubility of the film by 101.77 ± 7.47% and 26.51 ± 0.60%, respectively.	Incorporation of chitosan and ZnO into the film increased the peak decomposition temperature (Tp) to 197.88 ± 3.81 °C.	The antimicrobial rate is >99%, and it is effective against common food-borne pathogens. The DPPH/ABTS scavenging rate is >80%, delaying food oxidation.	Tomatoes remained fresh for 6 days at 25 °C, and at 4 °C for more than 10 days.	[86]
CDs	0.5%	Outer layer (GPC): gelatin-PVA-banana peel carbon dots Inner layer (CPC): corn starch-polyvinyl alcohol-cinnamon essential oil	The addition of CDs further enhanced the hydrogen bonds in the composite film (GPC film), but did not significantly change the mechanical proper-ties of the GPC film.	The hydrogen bond interaction between CDs and the film matrix prolongs the tortuous path of water molecules, showing better water vapor barrier ability. The strong UV absorption and reflection ability of carbon dots significantly reduces the UV transmittance of GP films.	In the range of 50–250 °C, the weight loss of the GPC film is lower than that of the GP film, and the residual weight is higher, indicating that CDs further improve its thermal stability.	After adding 0.5% CDs, the DPPH radical scavenging rate of the composite film (GPC) increased to 14.85%, while the ABTS radical scavenging rate increased significantly to 77.68%.	At 25 °C, the shelf life of mangoes is extended from 2 days to 8 days. After 28 days, the retention rate of capsanthin in spicy essential oil microcapsule powders is effectively increased.	[87]
CDs	-	Gelatin, *Sophora japonica* extract (95% rutin)-derived carbon dots (R-CDs)	After adding R-CDs, the TS of the gelatin film first increased and then decreased (1% optimal), and the EB continued to increase (1.5% to 358%).	Compared with pure gelatin film, the UV transmittance is reduced to less than 5% after adding R-CDs.	-	With the increase in concentration, R-CDs exhibited enhanced effects on the reduction rate of KMnO_4_, DPPH scavenging activity, and O_2_^•−^ scavenging activity.	When coated strawberries are stored at room temperature for 8 days, their rot rate is reduced from 75% to 4.17%, the weight loss rate is reduced from 45.47% to 29.63%, their hardness is higher and their appearance is full.	[88]

Notes: “-” indicates that the corresponding concentration or property of nanomaterials was not reported in the references.

## Data Availability

Data will be made available upon request.

## References

[1] Ahmed M.W., Haque M.A., Mohibbullah M., Khan M.S.I., Islam M.A., Mondal M.H.T., Ahmmed R. (2022). A review on active packaging for quality and safety of foods: Current trends, applications, prospects and challenges. Food Packag. Shelf Life.

[2] Wu X., Liu P., Shi H., Wang H., Huang H., Shi Y., Gao S. (2021). Photo aging and fragmentation of polypropylene food packaging materials in artificial seawater. Water Res..

[3] Wang K., Yang X., Liang J., Rong Y., Zhao W., Ding J., Liu Y., Liu Q. (2024). Preparation, characterization, antimicrobial evaluation, and grape preservation applications of polyvinyl alcohol/gelatin composite films containing zinc oxide quaternized chitosan nanoparticles. Int. J. Biol. Macromol..

[4] Song Z., Zang Z., Cao Y., Ma Y., Li B., Han L., Yu Q. (2025). Tapioca starch/konjac gum-based composite film incorporated with nanoliposomes encapsulated grape seed oil: Structure, functionality, controlled release and its preservation role for chilled mutton. Food Chem..

[5] Li L., Guo W., Wang L., Cheng S., Cheng H. (2025). Chitosan derived nano-selenium based coatings for postharvest safety of cherry tomato. LWT.

[6] Bu N., Huang L., Cao G., Lin H., Pang J., Wang L., Mu R. (2022). Konjac glucomannan/Pullulan films incorporated with cellulose nanofibrils-stabilized tea tree essential oil Pickering emulsions. Colloids Surf. A Physicochem. Eng. Asp..

[7] Cheng H., Xu H., Julian McClements D., Chen L., Jiao A., Tian Y., Miao M., Jin Z. (2022). Recent advances in intelligent food packaging materials: Principles, preparation and applications. Food Chem..

[8] Jafarzadeh S., Jafari S.M. (2021). Impact of metal nanoparticles on the mechanical, barrier, optical and thermal properties of biodegradable food packaging materials. Crit. Rev. Food Sci. Nutr..

[9] Lau W.W.Y., Shiran Y., Bailey R.M., Cook E., Stuchtey M.R., Koskella J., Velis C.A., Godfrey L., Boucher J., Murphy M.B. (2020). Evaluating scenarios toward zero plastic pollution. Science.

[10] Behrooznia Z., Nourmohammadi J. (2024). Polysaccharide-based materials as an eco-friendly alternative in biomedical, environmental, and food packaging. Giant.

[11] Zhang W., Rhim J.-W. (2022). Titanium dioxide (TiO_2_) for the manufacture of multifunctional active food packaging films. Food Packag. Shelf Life.

[12] Ding J., Hui A., Wang W., Yang F., Kang Y., Wang A. (2021). Multifunctional palygorskite@ZnO nanorods enhance simultaneously mechanical strength and antibacterial properties of chitosan-based film. Int. J. Biol. Macromol..

[13] Youssef D.M., Alshubaily F.A., Tayel A.A., Alghuthaymi M.A., Al-Saman M.A. (2022). Application of nanocomposites from bees products and nano-selenium in edible coating for catfish fillets biopreservation. Polymers.

[14] Alizadeh-Sani M., Mohammadian E., Rhim J.-W., Jafari S.M. (2020). pH-sensitive (halochromic) smart packaging films based on natural food colorants for the monitoring of food quality and safety. Trends Food Sci. Technol..

[15] Siripatrawan U., Kaewklin P. (2018). Fabrication and characterization of chitosan-titanium dioxide nanocomposite film as ethylene scavenging and antimicrobial active food packaging. Food Hydrocoll..

[16] Zhang W., Ahari H., Zhang Z., Jafari S.M. (2023). Role of silica (SiO_2_) nano/micro-particles in the functionality of degradable packaging films/coatings and their application in food preservation. Trends Food Sci. Technol..

[17] Yu Y., Zhou J., Chen Q., Xie F., Zhang D., He Z., Cheng S., Cai J. (2024). Self-reinforced multifunctional starch nanocomposite film for litchi fruit postharvest preservation. Chem. Eng. J..

[18] Cheng K., Xu F., Du J., Li C., Wang Z., Zhang L., Wang X., Liu J. (2025). Enhancing the preservation of blueberry with a tough and biodegradable soy protein isolate-carboxymethyl chitosan film integrated with TiO_2_ nanotube arrays. Food Packag. Shelf Life.

[19] Guerra I.C., de Sousa T.L., de Farias P.M., Cappato L.P., de Freitas B.S.M., Romani V.P., Plácido G.R. (2023). Films and coatings from pequi mesocarp incorporated with nano-ZnO: Properties and capacity to increase mango shelf life. Ind. Crops Prod..

[20] Tran T.H., Le X.C., Tran T.N.M., Nguyen N.T.T., Pham B.N., Vu D. (2023). Nano selenium–alginate edible coating extends hydroponic strawberry shelf life and provides selenium fortification as a micro-nutrient. Food Biosci..

[21] Mishra P., Jain T., Motiani M., Sarkar R., Shaw A. (2017). Have green, pay more: An empirical investigation of consumer’s attitude towards green packaging in an emerging economy. Essays on Sustainability and Management: Emerging Perspectives.

[22] Kumar V.A., Hasan M., Mangaraj S., Pravitha M., Verma D.K., Srivastav P.P. (2022). Trends in edible packaging films and its prospective future in food: A review. Appl. Food Res..

[23] Aradmehr A., Javanbakht V. (2020). A novel biofilm based on lignocellulosic compounds and chitosan modified with silver nanoparticles with multifunctional properties: Synthesis and characterization. Colloids Surf. A Physicochem. Eng. Asp..

[24] Li J., Zhuang S. (2020). Antibacterial activity of chitosan and its derivatives and their interaction mechanism with bacteria: Current state and perspectives. Eur. Polym. J..

[25] Adiletta G., Di Matteo M., Petriccione M. (2021). Multifunctional Role of Chitosan Edible Coatings on Antioxidant Systems in Fruit Crops: A Review. Int. J. Mol. Sci..

[26] Liu X., Xu Y., Liao W., Guo C., Gan M., Wang Q. (2023). Preparation and characterization of chitosan/bacterial cellulose composite biodegradable films combined with curcumin and its application on preservation of strawberries. Food Packag. Shelf Life.

[27] Beji E., Keshk S.M.A.S., Douiri S., Charradi K., Ben Hassen R., Gtari M., Attia H., Ghorbel D. (2023). Bioactive film based on chitosan incorporated with cellulose and aluminum chloride for food packaging application: Fabrication and characterization. Food Biosci..

[28] Luo Q., Hossen M.A., Zeng Y., Dai J., Li S., Qin W., Liu Y. (2022). Gelatin-based composite films and their application in food packaging: A review. J. Food Eng..

[29] Haghighi H., De Leo R., Bedin E., Pfeifer F., Siesler H.W., Pulvirenti A. (2019). Comparative analysis of blend and bilayer films based on chitosan and gelatin enriched with LAE (lauroyl arginate ethyl) with antimicrobial activity for food packaging applications. Food Packag. Shelf Life.

[30] Zhang W., Azizi-Lalabadi M., Jafarzadeh S., Jafari S.M. (2023). Starch-gelatin blend films: A promising approach for high-performance degradable food packaging. Carbohydr. Polym..

[31] Hossen M.A., Shimul I.M., Sameen D.E., Rasheed Z., Dai J., Li S., Qin W., Tang W., Chen M., Liu Y. (2024). Essential oil–loaded biopolymeric particles on food industry and packaging: A review. Int. J. Biol. Macromol..

[32] Sun C., Wang Y., Luan Q., Chen H. (2024). Preparation and properties of edible active films of gelatin/carboxymethyl cellulose loaded with resveratrol. Int. J. Biol. Macromol..

[33] Xu X., Dai D., Yan H., Du J., Zhang Y., Chen T. (2025). Enhancing mechanical and blocking properties of gelatin films using zein-quercetin nanoparticle and applications for strawberry preservation. Food Chem..

[34] Pawar S.N., Edgar K.J. (2012). Alginate derivatization: A review of chemistry, properties and applications. Biomaterials.

[35] Kaczmarek B. (2020). Improving sodium alginate films properties by phenolic acid addition. Materials.

[36] Lin X., Zhang H., Guo X., Qin Y., Shen P., Peng Q. (2023). A novel sodium alginate-carnauba wax film containing calcium ascorbate: Structural properties and preservative effect on fresh-cut apples. Molecules.

[37] Riahi Z., Khan A., Rhim J.-W., Shin G.H., Kim J.T. (2025). Red pepper waste-derived carbon dots incorporated sodium alginate/gelatin composite films for bioactive fruit preservation. Int. J. Biol. Macromol..

[38] Zhang Y., Zhao W., Lin Z., Tang Z., Lin B. (2023). Carboxymethyl chitosan/sodium alginate hydrogel films with good biocompatibility and reproducibility by in situ ultra-fast crosslinking for efficient preservation of strawberry. Carbohydr. Polym..

[39] Cao S., Wang S., Wang Q., Lin G., Niu B., Guo R., Yan H., Wang H. (2023). Sodium alginate/chitosan-based intelligent bilayer film with antimicrobial activity for pork preservation and freshness monitoring. Food Control.

[40] Behera L., Mohanta M., Thirugnanam A. (2022). Intensification of yam-starch based biodegradable bioplastic film with bentonite for food packaging application. Environ. Technol. Innov..

[41] Liu Z., Han J.H. (2005). 19—Edible films and coatings from starches. Innovations in Food Packaging.

[42] Podshivalov A., Zakharova M., Glazacheva E., Uspenskaya M. (2017). Gelatin/potato starch edible biocomposite films: Correlation between morphology and physical properties. Carbohydr. Polym..

[43] Cheetham N.W.H., Tao L. (1998). Variation in crystalline type with amylose content in maize starch granules: An X-ray powder diffraction study. Carbohydr. Polym..

[44] Thakur R., Pristijono P., Scarlett C.J., Bowyer M., Singh S.P., Vuong Q.V. (2019). Starch-based films: Major factors affecting their properties. Int. J. Biol. Macromol..

[45] Villas-Boas F., Franco C.M.L. (2016). Effect of bacterial β-amylase and fungal α-amylase on the digestibility and structural characteristics of potato and arrowroot starches. Food Hydrocoll..

[46] Kaur K., Jindal R., Maiti M., Mahajan S. (2019). Studies on the properties and biodegradability of PVA/Trapa natans starch (N-st) composite films and PVA/N-st-g-poly (EMA) composite films. Int. J. Biol. Macromol..

[47] Kaur M., Sharma S., Kalia A., Jawandha S.K. (2024). Maize starch-PVA nanocomposite biodegradable antimicrobial packaging films for enhancement of shelf-life of Agaricus bisporus. Sustain. Mater. Technol..

[48] Luo D., Xie Q., Gu S., Xue W. (2022). Potato starch films by incorporating tea polyphenol and MgO nanoparticles with enhanced physical, functional and preserved properties. Int. J. Biol. Macromol..

[49] Zdanowicz M., Markowska-Szczupak A., Spychaj T. (2020). Carboxymethyl Starch/Medium Chain Fatty Acid Compositions: Rheological Changes During Storage and Selected Film Properties. Starch.

[50] Zidan N., Albalawi M.A., Alalawy A.I., Al-Duais M.A., Alzahrani S., Kasem M., Tayel A.A., Nagib R.M. (2023). Active and smart antimicrobial food packaging film composed of date palm kernels extract loaded carboxymethyl chitosan and carboxymethyl starch composite for prohibiting foodborne pathogens during fruits preservation. Eur. Polym. J..

[51] Xie D., Liang Y., Zheng X., Zhu M., Huang G., Lin B. (2024). High-strength, antifogging and antibacterial ZnO/carboxymethyl starch/chitosan film with unique “Steel Wire Mesh” structure for strawberry preservation. Int. J. Biol. Macromol..

[52] Ezati P., Riahi Z., Rhim J.-W. (2022). CMC-based functional film incorporated with copper-doped TiO_2_ to prevent banana browning. Food Hydrocoll..

[53] Guo H., Bai J., Jin X., Liu H., Wu D., Gan R., Gao H. (2024). Innovative edible films for food preservation: Combining pectin and flavonoids from citrus peels with soy protein isolates. LWT.

[54] Li X., He J., Zhang W., Khan M.R., Ahmad N., Tian W. (2024). Pectin film fortified with zein nanoparticles and Fe^3+^-Encapsulated propolis extract for enhanced fruit preservation. Food Hydrocoll..

[55] Zhou C., Li L., Li D., Zhang R., Hu S., Zhong K., Yan B. (2024). Hyaluronic acid-based multifunctional bio-active coating integrated with cinnamaldehyde/hydroxypropyl-β-cyclodextrin inclusion complex for fruit preservation. Int. J. Biol. Macromol..

[56] Al-Hilifi S.A., Al-Ali R.M., Dinh L.N.M., Yao Y., Agarwal V. (2024). Development of hyaluronic acid based polysaccharide-protein composite edible coatings for preservation of strawberry fruit. Int. J. Biol. Macromol..

[57] Rastogi A., Sahoo S., Bandyopadhyay T.K., Mukherjee R., Banerjee R. (2022). Detailed morphological and kinetic studies of cellulose biosynthesis from *Leifsonia soli*. Polymer.

[58] Shi Z., Zhang Y., Phillips G.O., Yang G. (2014). Utilization of bacterial cellulose in food. Food Hydrocoll..

[59] Li J., Zhang F., Zhong Y., Zhao Y., Gao P., Tian F., Zhang X., Zhou R., Cullen P.J. (2022). Emerging food packaging applications of cellulose nanocomposites: A review. Polymers.

[60] Lindman B., Karlström G., Stigsson L. (2010). On the mechanism of dissolution of cellulose. J. Mol. Liq..

[61] Liu Y., Ahmed S., Sameen D.E., Wang Y., Lu R., Dai J., Li S., Qin W. (2021). A review of cellulose and its derivatives in biopolymer-based for food packaging application. Trends Food Sci. Technol..

[62] Hadimani S., Supriya D., Roopa K., Soujanya S.K., Rakshata V., Netravati A., Akshayakumar V., De Britto S., Jogaiah S. (2023). Biodegradable hybrid biopolymer film based on carboxy methyl cellulose and selenium nanoparticles with antifungal properties to enhance grapes shelf life. Int. J. Biol. Macromol..

[63] Zheng M., Chen J., Tan K.B., Chen M., Zhu Y. (2022). Development of hydroxypropyl methylcellulose film with xanthan gum and its application as an excellent food packaging bio-material in enhancing the shelf life of banana. Food Chem..

[64] Chen J., Zheng M., Tan K.B., Lin J., Chen M., Zhu Y. (2022). Development of xanthan gum/hydroxypropyl methyl cellulose composite films incorporating tea polyphenol and its application on fresh-cut green bell peppers preservation. Int. J. Biol. Macromol..

[65] Zheng M., Chen M., Xiao R., Feng J., Su H., Lin F., Chen J., Tan K.B., Zhu Y. (2025). Xanthan gum/hydroxypropyl methylcellulose/carbon quantum dots composite to enhance mango shelf life by triggering metacaspase-dependent apoptosis in *Colletotrichum gloeosporioides*. Int. J. Biol. Macromol..

[66] Mizielińska M., Bartkowiak A. (2024). The Influence of Zinc Oxide and Zinc Stearate on the Antimicrobial Activity of Coatings Containing Raspberry and Chokeberry Extracts. Molecules.

[67] Mellinas C., Ramos M., Jiménez A., Garrigós M.C. (2020). Recent trends in the use of pectin from agro-waste residues as a natural-based biopolymer for food packaging applications. Materials.

[68] Reichembach L.H., Lúcia de Oliveira Petkowicz C. (2021). Pectins from alternative sources and uses beyond sweets and jellies: An overview. Food Hydrocoll..

[69] Huang J., Hu Z., Hu L., Li G., Yao Q., Hu Y. (2021). Pectin-based active packaging: A critical review on preparation, physical properties and novel application in food preservation. Trends Food Sci. Technol..

[70] Vázquez J.A., Rodríguez-Amado I., Montemayor M.I., Fraguas J., González M.D., Murado M.A. (2013). Chondroitin sulfate, hyaluronic acid and chitin/chitosan production using marine waste sources: Characteristics, applications and eco-friendly processes: A review. Mar. Drugs.

[71] Kweon D.K., Han J.A. (2023). Development of hyaluronic acid-based edible film for alleviating dry mouth. Food Sci. Hum. Wellness.

[72] Ke C., Sun L., Qiao D., Wang D., Zeng X. (2011). Antioxidant acitivity of low molecular weight hyaluronic acid. Food Chem. Toxicol..

[73] Ouzakar S., Skali Senhaji N., Saidi M.Z., El Hadri M., El Baaboua A., El Harsal A., Abrini J. (2023). Antibacterial and antifungal activity of zinc oxide nanoparticles produced by *Phaeodactylum tricornutum* culture supernatants and their potential application to extend the shelf life of sweet cherry (*Prunus avium* L.). Biocatal. Agric. Biotechnol..

[74] Li H., Li F., Wang L., Sheng J., Xin Z., Zhao L., Xiao H., Zheng Y., Hu Q. (2009). Effect of nano-packing on preservation quality of Chinese jujube (*Ziziphus jujuba* Mill. var. inermis (Bunge) Rehd). Food Chem..

[75] Cano L., Pollet E., Avérous L., Tercjak A. (2017). Effect of TiO_2_ nanoparticles on the properties of thermoplastic chitosan-based nano-biocomposites obtained by mechanical kneading. Compos. Part A Appl. Sci. Manuf..

[76] Shoeibi S., Mashreghi M. (2017). Biosynthesis of selenium nanoparticles using *Enterococcus faecalis* and evaluation of their antibacterial activities. J. Trace Elem. Med. Biol..

[77] Huang B., Zhang J., Hou J., Chen C. (2003). Free radical scavenging efficiency of Nano-Se in vitro. Free Radic. Biol. Med..

[78] Cheng H., Wang L., Jia S., Wang L., Cheng S., Lu Y., Li L. (2025). Green synthesis of selenium nanoparticles by grape seed extract synergized with ascorbic acid: Preparation optimization, structural characterization, and functional activity. Foods.

[79] Yao B., Huang H., Liu Y., Kang Z. (2019). Carbon dots: A small conundrum. Trends Chem..

[80] Xu L., Zheng Z., Fang L., Zhou J., Zheng X., Gao Q., Xu C. (2025). Characterization of cellulose-derived carbon dots/polyvinyl alcohol films and their application in blueberry preservation. Int. J. Biol. Macromol..

[81] Zhang X., Li Y., Li Y., Li C. (2025). Development of a high-barrier, antimicrobial, and pH-sensitive nanocomposite film based on gellan gum/sodium carboxymethylcellulose/sodium alginate with nano-SiO(2) for strawberry preservation and monitoring. Int. J. Biol. Macromol..

[82] Kang L., Liang Q., Liu Y., Rashid A., Qayum A., Zhou C., Han X., Ren X., Chi Z., Chi R. (2024). Preparation technology and preservation mechanism of novel Ag NPs-loaded ZIF-67 packaging film. Food Packag. Shelf Life.

[83] Xu W., Xie W., Huang X., Chen X., Huang N., Wang X., Liu J. (2017). The graphene oxide and chitosan biopolymer loads TiO_2_ for antibacterial and preservative research. Food Chem..

[84] Wang Y., Zhang Y., Ma Y., Liu J., Zhang R., Zhao J. (2025). Preparation and application of chitosan/nano-TiO_2_/daisy essential oil composite films in the preservation of Actinidia arguta. Food Chem. X.

[85] Nguyen S.V., Lee B.-K. (2022). PVA/CNC/TiO_2_ nanocomposite for food-packaging: Improved mechanical, UV/water vapor barrier, and antimicrobial properties. Carbohydr. Polym..

[86] Wang Y., Li R., Yang X., Li L. (2025). Multifunctional film of soy protein nanofiber/chitosan/zinc oxide for tomato postharvest freshness preservation. Food Hydrocoll..

[87] Chen K., Zhang M., Bhandari B., Deng D. (2024). 3D printed cinnamon essential oil/banana peel carbon dots loaded corn starch/gelatin bilayer film with enhanced functionality for food packaging application. Food Chem..

[88] Guo B., Liu G., Ye W., Xu Z., Li W., Zhuang J., Zhang X., Wang L., Lei B., Hu C. (2024). Multifunctional carbon dots reinforced gelatin-based coating film for strawberry preservation. Food Hydrocoll..

[89] Pan J.-N., Sun J., Shen Q.-J., Zheng X., Zhou W.-W. (2025). Fabrication, properties, and improvement strategies of edible films for fruits and vegetables preservation: A comprehensive review. Food Innov. Adv..

[90] Zhang X., Xiao G., Wang Y., Zhao Y., Su H., Tan T. (2017). Preparation of chitosan-TiO_2_ composite film with efficient antimicrobial activities under visible light for food packaging applications. Carbohydr. Polym..

[91] Flaker C.H.C., Lourenço R.V., Bittante A.M.Q.B., Sobral P.J.A. (2015). Gelatin-based nanocomposite films: A study on montmorillonite dispersion methods and concentration. J. Food Eng..

[92] Shah Y.A., Bhatia S., Al-Harrasi A., Tarahi M., Almasi H., Chawla R., Ali A.M.M. (2024). Insights into recent innovations in barrier resistance of edible films for food packaging applications. Int. J. Biol. Macromol..

[93] Motelica L., Ficai D., Petrisor G., Oprea O.-C., Trușcǎ R.-D., Ficai A., Andronescu E., Hudita A., Holban A.M. (2024). Antimicrobial Hydroxyethyl-Cellulose-Based Composite Films with Zinc Oxide and Mesoporous Silica Loaded with Cinnamon Essential Oil. Pharmaceutics.

[94] La D.D., Nguyen-Tri P., Le K.H., Nguyen P.T.M., Nguyen M.D., Vo A.T.K., Nguyen M.T.H., Chang S.W., Tran L.D., Chung W.J. (2021). Effects of antibacterial ZnO nanoparticles on the performance of a chitosan/gum arabic edible coating for post-harvest banana preservation. Prog. Org. Coat..

[95] Mouzahim M.E., Eddarai E.M., Eladaoui S., Guenbour A., Bellaouchou A., Zarrouk A., Boussen R. (2023). Effect of Kaolin clay and *Ficus carica* mediated silver nanoparticles on chitosan food packaging film for fresh apple slice preservation. Food Chem..

[96] Cui N., Li Y., Du H., Liang L., Wen Y., Zhu Z. (2025). Chitosan-based film with enhanced gas permeability and antimicrobial property for fresh fruit preservation. Food Res. Int..

[97] Surendhiran D., Roy V.C., Park J.-S., Chun B.-S. (2022). Fabrication of chitosan-based food packaging film impregnated with turmeric essential oil (TEO)-loaded magnetic-silica nanocomposites for surimi preservation. Int. J. Biol. Macromol..

[98] Ediyilyam S., George B., Shankar S.S., Dennis T.T., Wacławek S., Černík M., Padil V.V.T. (2021). Chitosan/Gelatin/Silver Nanoparticles Composites Films for Biodegradable Food Packaging Applications. Polymers.

[99] Shan P., Wang K., Sun F., Li Y., Sun L., Li H., Peng L. (2024). Humidity-adjustable functional gelatin hydrogel/ethyl cellulose bilayer films for active food packaging application. Food Chem..

[100] Yang S., Xu Y., Pan A., Li P., Yang W., Sun Q., Zhang X., Xu Y. (2025). Preparation of a high-strength, antibacterial, and biodegradable bio-based film through amide crosslinking and nano-reinforcement for fruit packaging. Chem. Eng. J..

[101] Sabzevari S., Farrokhzad H., Poorkhalil A. (2025). Development of citric acid-crosslinked carboxymethyl cellulose/chitosan hydrogel films reinforced with ZnO nanoparticles for active broccoli packaging. Food Packag. Shelf Life.

[102] Khan A., Riahi Z., Zhang W., Priyadarshi R., Kumar J.V., Rhim J.-W., Kim J.T., Chang Y.H., Benjakul S. (2025). Health and safety aspects of carbon dots and their sustainable applications in food packaging and preservation. Sustain. Mater. Technol..

[103] Jiang Z., Feng J., Dai Y., Yu W., Bai S., Bai C., Tu Z., Guo P., Liao T., Qiu L. (2025). Preparation of a biodegradable packaging film by konjac glucomannan/sodium alginate reinforced with nitrogen-doped carbon quantum dots from crayfish shell for crayfish meat preservation. Int. J. Biol. Macromol..

[104] Yan Z., Chen H., Li H., Xia B., Wang K. (2025). Preparation of intelligent and active κ-carrageenan-based packaging film incorporating bayberry anthocyanins and iron-doped carbon dots for fresh shrimp preservation. Carbohydr. Polym..

[105] EFSA Panel on Food Contact Material, Enzymes, Flavourings and Processing Aids (CEF) (2014). Statement on the safety assessment of the substance silicon dioxide, silanated, FCM Substance No 87 for use in food contact materials. EFSA J..

[106] Ton-That P., Dinh T.A., Thanh Gia-Thien H., Van Minh N., Nguyen T., Huynh K.P.H. (2025). Novel packaging chitosan film decorated with green-synthesized nanosilver derived from dragon fruit stem. Food Hydrocoll..

[107] EFSA Scientific Committee (2011). Guidance on the risk assessment of the application of nanoscience and nanotechnologies in the food and feed chain. EFSA J..

[108] Lambré C., Barat Baviera J.M., Bolognesi C., Chesson A., Cocconcelli P.S., Crebelli R., Gott D.M., Grob K., Lampi E., EFSA Panel on Food Contact Materials, Enzymes and Processing Aids (CEP) (2021). Safety assessment of the substance silver nanoparticles for use in food contact materials. EFSA J..

[109] Gao Q., Feng Z., Wang J., Zhao F., Li C., Ju J. (2025). Application of nano-ZnO in the food preservation industry: Antibacterial mechanisms, influencing factors, intelligent packaging, preservation film and safety. Crit. Rev. Food Sci. Nutr..

[110] Bumbudsanpharoke N., Choi J., Park H.J., Ko S. (2019). Zinc migration and its effect on the functionality of a low density polyethylene-ZnO nanocomposite film. Food Packag. Shelf Life.

[111] Hackenberg S., Scherzed A., Technau A., Kessler M., Froelich K., Ginzkey C., Koehler C., Burghartz M., Hagen R., Kleinsasser N. (2011). Cytotoxic, genotoxic and pro-inflammatory effects of zinc oxide nanoparticles in human nasal mucosa cells in vitro. Toxicol. Vitr..

[112] Wang Z., Zhang R., Yang C., Yi H., Yang W., Hu Y. (2025). A pH-responsive and antibacterial double-layer smart nanofiber film (PCL/AlPc-PCL/PVA/CS/ACN) for pork preservation monitoring. Food Chem..

[113] Dodero A., Alloisio M., Vicini S., Castellano M. (2020). Preparation of composite alginate-based electrospun membranes loaded with ZnO nanoparticles. Carbohydr. Polym..

[114] Banitaba S.N., Khoshnama A., Poursharifi N., Nasari M., Semnani D., Jafari M. (2021). Fabrication and Characterization of the Electrospun Polyvinyl Alcohol Nanofibers Incorporated with the Extracted Fruit Peel Pectin and Zinc Oxide Nanoparticles. Mater. Perform. Charact..

[115] He Y., Zhong T., Liu Y., Wan M., Sun L., Zhao Y., Wang Z. (2024). Development of a multifunctional active food packaging membrane based on electrospun polyvinyl alcohol/chitosan for preservation of fruits. Int. J. Biol. Macromol..

[116] Oishi Y., Nakaya M., Matsui E., Hotta A. (2015). Structural and mechanical properties of cellulose composites made of isolated cellulose nanofibers and poly(vinyl alcohol). Compos. Part A Appl. Sci. Manuf..

[117] Fabra M.J., López-Rubio A., Ambrosio-Martín J., Lagaron J.M. (2016). Improving the barrier properties of thermoplastic corn starch-based films containing bacterial cellulose nanowhiskers by means of PHA electrospun coatings of interest in food packaging. Food Hydrocoll..

[118] Khodaei D., Oltrogge K., Hamidi-Esfahani Z. (2020). Preparation and characterization of blended edible films manufactured using gelatin, tragacanth gum and, Persian gum. LWT.

[119] Lian Z., Zhang Y., Zhao Y. (2016). Nano-TiO_2_ particles and high hydrostatic pressure treatment for improving functionality of polyvinyl alcohol and chitosan composite films and nano-TiO_2_ migration from film matrix in food simulants. Innov. Food Sci. Emerg. Technol..

[120] do Val Siqueira L., Arias C.I.L.F., Maniglia B.C., Tadini C.C. (2021). Starch-based biodegradable plastics: Methods of production, challenges and future perspectives. Curr. Opin. Food Sci..

[121] Cheng H., Chen L., McClements D.J., Yang T., Zhang Z., Ren F., Miao M., Tian Y., Jin Z. (2021). Starch-based biodegradable packaging materials: A review of their preparation, characterization and diverse applications in the food industry. Trends Food Sci. Technol..

[122] Herniou J.C., Mendieta J.R., Gutiérrez T.J. (2019). Characterization of biodegradable/non-compostable films made from cellulose acetate/corn starch blends processed under reactive extrusion conditions. Food Hydrocoll..

[123] Redfearn H.N., Warren M.K., Goddard J.M. (2023). Reactive Extrusion of Nonmigratory Active and Intelligent Packaging. ACS Appl. Mater. Interfaces.

[124] Feng N., Zhang J., Tian J., Zhang Y., Li M., Guo X., Han Q., Wang Y., Gao A., Wang Y. (2025). Preserving fruit freshness with amyloid-like protein coatings. Nat. Commun..

[125] Xu W., Jia X., Yang M., Peng Z., Chen H., Zhang X., Wei B. (2025). Tea polyphenol self-assembly nanocomposite coating for fruit preservation. ACS Nano.

[126] Wang L., Wang L., Gong H., Li L., Lu Y., Cheng S., Cheng H. (2025). Antimicrobial SA/GL/SeNPs nanocomposite film: Fabrication and preservation efficacy in prolonging milk jujube shelf-life. J. Future Foods.

[127] Li S., Jiang Y., Zhou Y., Li R., Jiang Y., Alomgir Hossen M., Dai J., Qin W., Liu Y. (2022). Facile fabrication of sandwich-like anthocyanin/chitosan/lemongrass essential oil films via 3D printing for intelligent evaluation of pork freshness. Food Chem..

[128] Díaz-Montes E., Castro-Muñoz R. (2021). Edible films and coatings as food-quality preservers: An overview. Foods.

[129] Tavassoli-Kafrani E., Shekarchizadeh H., Masoudpour-Behabadi M. (2016). Development of edible films and coatings from alginates and carrageenans. Carbohydr. Polym..

[130] Priyadarshi R., Negi Y.S. (2017). Effect of varying filler concentration on Zinc oxide nanoparticle embedded chitosan films as potential food packaging material. J. Polym. Environ..

[131] Jo Y., Garcia C.V., Ko S., Lee W., Shin G.H., Choi J.C., Park S.-J., Kim J.T. (2018). Characterization and antibacterial properties of nanosilver-applied polyethylene and polypropylene composite films for food packaging applications. Food Biosci..

[132] Indumathi M.P., Saral Sarojini K., Rajarajeswari G.R. (2019). Antimicrobial and biodegradable chitosan/cellulose acetate phthalate/ZnO nano composite films with optimal oxygen permeability and hydrophobicity for extending the shelf life of black grape fruits. Int. J. Biol. Macromol..

[133] Suyatma N.E., Tighzert L., Copinet A., Coma V. (2005). Effects of hydrophilic plasticizers on mechanical, thermal, and surface properties of chitosan films. J. Agric. Food Chem..

[134] Rodríguez-Núñez J.R., Madera-Santana T.J., Sánchez-Machado D.I., López-Cervantes J., Soto Valdez H. (2014). Chitosan/hydrophilic plasticizer-based films: Preparation, physicochemical and antimicrobial properties. J. Polym. Environ..

[135] Zdanowicz M., Johansson C. (2016). Mechanical and barrier properties of starch-based films plasticized with two- or three component deep eutectic solvents. Carbohydr. Polym..

[136] Zdanowicz M., Johansson C. (2017). Impact of additives on mechanical and barrier properties of starch-based films plasticized with deep eutectic solvents. Starch.

[137] Jha P. (2020). Effect of plasticizer and antimicrobial agents on functional properties of bionanocomposite films based on corn starch-chitosan for food packaging applications. Int. J. Biol. Macromol..

[138] González-Torres B., Robles-García M.Á., Gutiérrez-Lomelí M., Padilla-Frausto J.J., Navarro-Villarruel C.L., Del-Toro-Sánchez C.L., Rodríguez-Félix F., Barrera-Rodríguez A., Reyna-Villela M.Z., Avila-Novoa M.G. (2021). Combination of sorbitol and glycerol, as plasticizers, and oxidized starch improves the physicochemical characteristics of films for food preservation. Polymers.

[139] Yang J., Zhang X., Chen L., Zhou X., Fan X., Hu Y., Niu X., Xu X., Zhou G., Ullah N. (2022). Antibacterial aerogels with nano-silver reduced in situ by carboxymethyl cellulose for fresh meat preservation. Int. J. Biol. Macromol..

[140] Ding K., Xie Y., Xu H., Xu S., Ge S., Li H., Chang X., Chen J., Wang R., Shan Y. (2024). Visible light-responsive TiO_2_-based hybrid nanofiller reinforced multifunctional chitosan film for effective fruit preservation. Food Chem..

[141] Xu J., Shi X., Liu M., Gu M., Dai X., Aziz T., Alharbi N.K., Mojally M.A., Al-Asmari F., Al-Joufi F.A. (2026). Bionanocomposite films constituted by chitosan and ZnO nanoparticles stabilized by tea polyphenols for mango preservation. Food Hydrocoll..

[142] Yan R., Liu M., Zeng X., Du Q., Wu Z., Guo Y., Tu M., Pan D. (2024). Preparation of modified chitosan-based nano-TiO_2_-nisin composite packaging film and preservation mechanism applied to chilled pork. Int. J. Biol. Macromol..

[143] Thanh Huong Q.T., Hoai Nam N.T., Duy B.T., An H., Hai N.D., Kim Ngan H.T., Ngan L.T., Le Hoai Nhi T., Yen Linh D.T., Khanh T.N. (2023). Structurally natural chitosan films decorated with Andrographis paniculata extract and selenium nanoparticles: Properties and strawberry preservation. Food Biosci..

[144] Almasi H., Jafarzadeh P., Mehryar L. (2018). Fabrication of novel nanohybrids by impregnation of CuO nanoparticles into bacterial cellulose and chitosan nanofibers: Characterization, antimicrobial and release properties. Carbohydr. Polym..

[145] Li D. (2025). Development and evaluation of titanium dioxide/chitosan nanocomposite coatings for enhanced food preservation and nutrient retention. Alex. Eng. J..

[146] Tao Y., Pan J., Yan S., Tang B., Zhu L. (2007). Tensile strength optimization and characterization of chitosan/TiO_2_ hybrid film. Mater. Sci. Eng. B.

[147] Wang H., Ding F., Ma L., Zhang Y. (2021). Edible films from chitosan-gelatin: Physical properties and food packaging application. Food Biosci..

[148] Cypriyana P.J.J., Saigeetha S., Angalene J.L.A., Samrot A.V., Kumar S.S., Ponniah P., Chakravarthi S. (2021). Overview on toxicity of nanoparticles, it’s mechanism, models used in toxicity studies and disposal methods—A review. Biocatal. Agric. Biotechnol..

[149] Espitia P.J.P., Soares N.d.F.F., Coimbra J.S.d.R., de Andrade N.J., Cruz R.S., Medeiros E.A.A. (2012). Zinc oxide nanoparticles: Synthesis, antimicrobial activity and food packaging applications. Food Bioprocess. Technol..

[150] Huang Z., Wu Z., Luo Z., Gong H., Li W., Jin P., Deng Z., Fang D. (2025). Development of temperature-sensitive and controlled-release PLA-based film for strawberry preservation. Food Packag. Shelf Life.

[151] Qu X., Han D., Chen Q., Ji G., Peng Y., Li S., Qin K., Ren S., Wang Y., Zhou H. (2025). Self-assembled multifunctional antimicrobial composite films of humidity-responsive pterostilbene@β-cyclodextrin inclusion complexes/carboxymethyl cellulose/zein for fruit preservation. Food Packag. Shelf Life.

[152] Wu Z., Zhu H., Zhang H., Erihemu, Li G., Qi W., Zhang P. (2025). Development and characterization of potato starch/chitosan/ε-polylysine composite films for enhancing cherry preservation under high-humidity conditions. Food Packag. Shelf Life.

[153] Gujar S. (2024). Food Packaging Market Size—By Material, by Product Type, by Packaging Type, by Technology, by Application Analysis, Share, Growth Forecast, 2025–2034.

